# Extra-Synaptic Functions of NRG1 Type I Signaling in Hippocampal **γ**-Oscillations

**DOI:** 10.1093/schbul/sbag168

**Published:** 2026-10-30

**Authors:** María Clara Soto-Bernardini, Sua Jeong, Hadiseh Hosseinnia, Bekir Altas, Hong Jun Rhee, Chungku Lee, Yvonne Schill, Ulrike Zeitschel, Tilmann Götze, Julia Hellriegel, Maria Lehning, Oscar Brenes, Jaime Fornaguera, José Aníbal Mora-Villalobos, Robert Fledrich, Ruth M Stassart, Jeong Seop Rhee, Markus H Schwab

**Affiliations:** Department of Neurogenetics, Max-Planck-Institute for Multidisciplinary Sciences, 37075 Göttingen, Lower Saxony, Germany; Paul Flechsig Institute, Centre for Neuropathology and Brain Research, Medical Faculty, Leipzig University, 04103 Leipzig, Saxony, Germany; Center for Research in Biotechnology (CIB), Costa Rica Institute of Technology (TEC), 30109 Cartago, Costa Rica; Department of Neurobiology, Max-Planck-Institute for Multidisciplinary Sciences, 37075 Göttingen, Lower Saxony, Germany; Paul Flechsig Institute, Centre for Neuropathology and Brain Research, Medical Faculty, Leipzig University, 04103 Leipzig, Saxony, Germany; Department of Neurobiology, Max-Planck-Institute for Multidisciplinary Sciences, 37075 Göttingen, Lower Saxony, Germany; Department of Neurobiology, Max-Planck-Institute for Multidisciplinary Sciences, 37075 Göttingen, Lower Saxony, Germany; Department of Neurobiology, Max-Planck-Institute for Multidisciplinary Sciences, 37075 Göttingen, Lower Saxony, Germany; Cellular Neurophysiology, Hannover Medical School, 30625 Hannover, Lower Saxony, Germany; Paul Flechsig Institute, Centre for Neuropathology and Brain Research, Medical Faculty, Leipzig University, 04103 Leipzig, Saxony, Germany; Department of Neurogenetics, Max-Planck-Institute for Multidisciplinary Sciences, 37075 Göttingen, Lower Saxony, Germany; Cellular Neurophysiology, Hannover Medical School, 30625 Hannover, Lower Saxony, Germany; Paul Flechsig Institute, Centre for Neuropathology and Brain Research, Medical Faculty, Leipzig University, 04103 Leipzig, Saxony, Germany; Paul Flechsig Institute, Centre for Neuropathology and Brain Research, Medical Faculty, Leipzig University, 04103 Leipzig, Saxony, Germany; Neuroscience Research Center (CIN), University of Costa Rica (UCR), 11501-2060 San José, Costa Rica; Department of Physiology, School of Medicine, University of Costa Rica (UCR), 11501 San José, Costa Rica; Neuroscience Research Center (CIN), University of Costa Rica (UCR), 11501-2060 San José, Costa Rica; National Center of Biotechnological Innovations (CENIBiot)/National Center of High Technology (CENAT), 1174-1200 San Jose, Costa Rica; Institute of Anatomy, Leipzig University, 04103 Leipzig, Saxony, Germany; Paul Flechsig Institute, Centre for Neuropathology and Brain Research, Medical Faculty, Leipzig University, 04103 Leipzig, Saxony, Germany; Department of Neurobiology, Max-Planck-Institute for Multidisciplinary Sciences, 37075 Göttingen, Lower Saxony, Germany; Department of Neurogenetics, Max-Planck-Institute for Multidisciplinary Sciences, 37075 Göttingen, Lower Saxony, Germany; Paul Flechsig Institute, Centre for Neuropathology and Brain Research, Medical Faculty, Leipzig University, 04103 Leipzig, Saxony, Germany

**Keywords:** schizophrenia, conditional transgenic mice, Kv2.1, ER-PM junctions

## Abstract

**Background and Hypothesis:**

The neurodevelopmental hypothesis of schizophrenia posits that early-acting genetic risk factors precipitate pathological neurodevelopmental processes that result in neural network dysfunctions during adolescence. Human gene variants of the epidermal growth factor-like ligand Neuregulin 1 (*NRG1*) and its tyrosine kinase receptor ErbB4 are putative risk factors in schizophrenia, and aberrant NRG1 signaling has been implicated in schizophrenia-relevant endophenotypes. However, insight into associated pathomechanisms remains limited.

**Study Design:**

We modeled embryonic glutamatergic neuron-specific NRG1 type I overexpression in a conditional transgenic mouse line to investigate the impact of early hyperactive NRG1 type I signaling on neuronal network functions in the hippocampus in vivo.

**Study Results:**

NRG1 type I transgenic mice display abnormal gamma-frequency network oscillations in the adolescent hippocampus, in the absence of neuroinflammation or changes in hippocampal morphology. Instead, we found extra-synaptic NRG1 type I accumulation, which was associated with ErbB4 hyperphosphorylation, an increased size and number of Kv2.1 potassium channel–marked endoplasmic reticulum (ER)-plasma membrane (PM) junctions, and a reduction of A-type potassium channel currents in pyramidal cells.

**Conclusions:**

Together, these findings suggest a model in which early-onset extra-synaptic NRG1 type I accumulation at ER-PM junctions causes sustained changes in neuronal excitability, ultimately contributing to deficits in neuronal network oscillations as observed in various neuropsychiatric disorders.

## Introduction

Schizophrenia is a highly heritable disorder, much of which is attributable to a large number of common genetic variants with small effect size.^1^ According to the neurodevelopmental hypothesis of schizophrenia, early genetic insults induce pathological processes in the developing brain. During adolescence this may result in disturbed neuronal networks that are more prone to produce psychotic symptoms, particularly when triggered by a second “hit”, eg, heightened psychological stress.^2^^,^^3^ This concept is supported by genome-wide association studies (GWAS) with human samples in which genetic risk was enriched in biological processes of neuronal development and synapse formation,^4^ as well as in regulatory DNA regions linked to transcriptional dysregulation of genes with neurodevelopmental functions.^5^^,^^6^ However, early-acting genetic risk factor–mediated pathomechanisms are largely unexplored, mostly due to the limited availability of suitable mouse models.

Human *NRG1* and *ERBB4* gene variants are putative risk factors in schizophrenia,^7–9^ and ErbB receptor signaling was identified as a schizophrenia–relevant signaling pathway in a protein network-based analysis of GWAS–derived genetic risk factors.^10^ While recent GWAS studies have not consistently replicated earlier findings, genetic, functional, and preclinical studies continue to support an important role for NRG1-ErbB4 signaling in psychosis-relevant endophenotypes, including neuroinflammation, synaptic and network dysfunctions, aberrant neuroimaging, and behavioral deficits.^11–14^

Altered oscillatory neuronal network activity has been repeatedly observed in neuropsychiatric disorders, including schizophrenia.^15^ NRG1 signaling to ErbB4, its main tyrosine kinase receptor in the central nervous system, is required for the development of glutamatergic, GABAergic, and dopaminergic neuronal networks,^16^ and altered **γ**-oscillation power in ErbB4 mouse mutants^17^^,^^18^ indicates a role of NRG1/ErbB4 signaling in regulating oscillatory neuronal network activity. Together with postmortem studies of increased expression of the NRG1 type I isoform and ErbB4 hyperphosphorylation in schizophrenia patients,^19^^,^^20^ these findings suggest that hyperactive NRG1/ErbB4 signaling could contribute to abnormal oscillatory activity in neuropsychiatric disorders. Notably, cortical expression of NRG1 type I peaks already during embryonic development,^21^ and embryonic, but not postnatal loss of ErbB4 in parvalbumin-expressing (PV^+^) interneurons was shown to disrupt patterned cortical activity in the adult brain.^22^ Together, these data indicate a pathomechanistic impact of early disturbances in NRG1 type I signaling on neuronal network functions, but this has not been studied in vivo.

In this study we show in a conditional transgenic mouse model that an embryonic onset of moderate NRG1 type I overexpression in glutamatergic neurons results in extra-synaptic NRG1 type I accumulation, increases the size and number of Kv2.1-marked ER-PM junctions, reduces A-type potassium channel currents and diminishes the power of hippocampal **γ**-oscillations at young adult stages.

## Materials and Methods

### Transgenic Mice

*STOP-Nrg1* mice were generated as previously described.^23^ Mice were maintained on a C57Bl/6N background in compliance with animal policies of the Max-Planck-Institute of Experimental Medicine and approved by the German Federal State of Lower Saxony.

Fluorescence of *Stop* cassette-encoded EGFP was used for routine phenotyping of *Stop-Nrg1* mice. Tail biopsies were examined under fluorescent light (488 nm) with a Leica MZ16F fluorescent stereomicroscope. For PCR-based genotyping, genomic DNA was isolated from tail biopsies according to the manufacturer’s manual (1^-Step^ Tissue & Cells genomic DNA isolation kit; nexttec™) and amplified using primer 1 (5′-GGTGGCTATAAAGAGGTCATCAG-3′) and primer 2 (5′-CTCCTGGCTTTTCATCTCTTTCAA-3′) (Suppl. Figure 1A). Genotyping of *NEX-Cre* mice was performed as described.^24^

### Biochemistry

Sucrose homogenization buffer (320 mM Sucrose, 10 mM Tris (pH 7.4), 1 mM NaHCO3 and 1 mM MgCl2) with protease and phosphatase inhibitors (Roche) was used for protein extraction as described.^25^ Proteins were separated on 8% SDS-polyacrylamide gels and blotted onto PVDF (Hybond-P, Invitrogen). Membranes were incubated with primary antibodies (Actin ms 1:1000 (Millipore), ErbB4 rb 1:1000 (Abcam), p-HER4/ErbB4 (Tyr1284) rb 1:1000 (Cell Signaling), AKT rb 1:1000 (Cell Signaling), p-AKT rb 1:1000 (Ser473) (Cell Signaling), p44/42 MAPK (Erk1/2) rb 1:1000 (Cell Signaling), p-p44/42 MAPK (Erk1/2) (Thr202/Tyr204) rb 1:1000 (Cell Signaling), HA ms 1:250 (Covance), Neuregulin-1a/b1/2 rb 1:500 (Santa Cruz)), overnight at 4°C. Membranes were washed 3× in 1× TBS-Tween and incubated in secondary antibodies (goat anti-ms-HRP, Dianova, 1:5000; goat anti-rb-HRP, Dianova, 1:5000) for 1 hour at RT, developed with Western Lightning Plus-ECL Oxidizing reagent plus and Enhanced luminol reagent plus solutions (Perkin Elmer Life Science, Inc.), and scanned using an Intas ChemoCam Imager. Densitometric analysis of band intensities was carried out using ImageJ software.

### Cell Culture, RNA Extraction, cDNA Synthesis, and qPCR

Hippocampal and cortical cultures were prepared from C57BL6/J mice at P1-2 according to an optimized protocol.^26^^,^^27^ In brief, tissue was isolated and dissociated neurons were plated onto sterilized 18 mm coverslips coated with 250 μg/ml PLL and 50 μg/ml PDL. After 1 hour incubation at 37°C, Neurobasal-A medium (Gibco) containing 2% (v/v) B27 supplement, 1% (v/v) GlutaMAX® and 0.2% (v/v) penicillin–streptomycin was added. Cultures were maintained at 37°C and 5% CO_2_. One-third of the medium was changed every second day. On DIV12, cultures were treated with 50 mM KCl or ddH_2_O (control) for 6 hours. Subsequently, mRNA was purified using the RNeasy® Plus Mini Kit (Qiagen) and transcribed into cDNA using the SuperScriptIII First-Strand Synthesis System (reverse transcription) (Life Technologies) according to the manufacturer’s protocol.

qPCR was performed using the standard protocol for relative quantification of the 7500 Fast Real-Time PCR (Applied Biosystems). 10 μl reactions were set up with 4 μl DNA (5 ng/μl), 0.1 μl sense primer (10 pM), 0.1 μl antisense primer (10 pM), 5 μl GoTaq qPCR Master Mix (Promega), and 0.8 μl ddH_2_O. The following primers were used: F5′-TTCTCACCACTGCTCCACAG-3′, R5′-CTCCACGTGTGGC-TCTCAT-3′ (*NRG2);* F5′-ACTCAGCCACAAACAACAGA-AAC-3′, R5′- GAAGCACTCGCCTCCATT-3′ (*NRG1* type III); F5′-GGAAGGGCAAGAAGAAGGAC -3′, R5′-CCTGGCTTT-TCATCTCTTTCA -3′ (*NRG1* type I); F5′-CTGCTCTTTCCCA-GACGAGG-3′, R5′-AAGGCCACTTATCACCAGCC-3′ (β-actin). To assess the expression of *NRG1* type II, 5 primer pairs were used F5′-CTGACAACTGGACCCTACTC-3′, R5′-TTTCACAGCCCACACCTG-3′ (Makinodan et al 2012), F5′- CCTCGTTTCCTCCGCTG-3′, R5′- GACTCCTGGCTTTTCA-TCTCTT-3′, F5′-GGGGGCTTGAAGAAGGACTC-3′, R5′-ACCTCCTTCTTGAGGTTGCG-3′, F5′- GAAGGACTCGCTA-CTCACCG-3′, R5′-CGGCTGACCTCCTTCTTGAG-3′, F5′-CGCCCTATCTGGTGAAGGTG-3′, R5′-CGGTGAGTAGCG-AGTCCTTC-3′. Ct values from quadruplicates were analyzed, and outliers were eliminated when the standard deviation was higher than to 0.5. Comparative quantification analysis was performed by the ΔΔCt method. The fold difference in expression (2-ΔΔCt) was determined. T-students were performed to compare the expression of each gene in treated cells and controls.

#### Immunocytochemistry

Hippocampal primary neurons cultured on PLL-coated coverslips, including KCl-treated and untreated control conditions, were fixed with 4% PFA for 20 minutes at RT, then washed three times with 1× PBS. Cells were permeabilized with 100% methanol for 5 minutes and incubated in blocking buffer (2% bovine-serum albumin, 2% horse-serum, 0.1% gelatin) 1 hour at RT. Coverslips were incubated overnight at 4°C with primary antibodies (Neurogranin, mouse, 1:500, Novusbio; Parvalbumin, mouse, 1:500, Swant) diluted in 10% blocking solution, followed by three 1× PBS washes. Afterwards, coverslips were incubated with secondary antibodies diluted in 5% blocking solution (αmouse-Cy3, Dianova; αrabbit-Alexa488, 1:750, Invitrogen) together with DAPI (300 nm) for 2 hours at RT. After final washes, coverslips were mounted with Aqua-Poly/Mount (Polysciences, Inc.) and stored at 4°C. Images were acquired using a Leica TCS SP2 (Leica Microsystems GmbH, Wetzlar, Germany).

### Histology and Immunohistochemistry

Paraplast embedding of brain tissue and fluorescent immunostaining was performed as described.^28^ Cryo- and vibratome sections were blocked and permeabilized with 5 % horse-serum, 0.1 % Triton X-100 in 1× PBS for 30 minutes at RT. Primary antibodies (αCre, rabbit, 1:1000, Covance; αGFAP, rabbit, 1:500, Dako; αGFP, goat, 1:500, Rockland; αHA, mouse, 1:100, Biolegend; αHA, chicken, 1:100, Sigma Aldrich; αHA, rabbit, Cell Signaling, 1:400; αNeuN, mouse, 1:100, αChemicon; NRG1, rabbit, 1:100, Santa Cruz; αOlig2, rabbit, 1:200, John Alberta, Harvard; αKv2.1, mouse, 1:500, Abcam; IBA, rabbit, 1:700, Wako; VGlut, guinea pig, 1:100, Synaptic Systems; PSD95 mouse, 1:500, Neuromab; Gephyrin, mouse, 1:500, Synaptic Systems; Synaptophysin, mouse, 1:1000; Synaptic Systems; SERT, goat, 1:100, Santa Cruz; GM130, ms, 1:200, BD Biosciences; ChAT, chicken, 1:500, Aves Labs; Parvalbumin, rabbit, 1:200, Swant) were diluted in blocking solution and incubated overnight at 4°C. After three washing steps in 1× PBS at RT secondary antibodies diluted in blocking solution (αchicken-cy3, 1:1000; αchicken-Alexa488, 1:1000; αgoat-Alexa488, 1:500; αmouse-Alexa555, 1:500; αmouse-DL649, 1:250; αmouse-Cy2, 1:100; αmouse-Cy3, 1:1000; αmouse-Alexa 488, 1:1000; αrabbit-Alexa488, 1:100; αrabbit-Alexa555, 1:500; αrabbit-Cy2, 1:100; αrabbit-Cy3, 1:1000; αrabbit-Cy5, 1:250; αrabbit-DL649, 1:250; α-guinea pig-cy3 1:1000, α-guinea pig-cy5 1:250 from Dianova and Molecular Probes) and DAPI (300 nM) were incubated for 2 hours at RT. Slides were washed three times in 1× PBS (protected from light), mounted in Aqua-Poly/Mount (Polysciences, Inc.) and stored at 4°C.

Chromogenic immunostaining on paraplast embedded brain tissue was performed as for fluorescent immunostaining, except for inactivation of endogenous peroxidases by 5 minutes incubation with 3 % hydrogen peroxide prior to blocking. Sections were incubated overnight at 4°C with primary antibodies (GFAP rb 1:500 (Dako), IBA1 rb 1:700 (Wako), NeuN ms 1:100 (Chemicon), Parvalbumin 28 rb 1:200 (Swant)), followed by secondary antibody (biotinylated, Dako LSAB2) and streptavidin conjugated to Horseradish Peroxidase (Dako LSAB2). Stainings were developed by addition of DAB substrate solution, counterstained with Hematoxylin, dehydrated in an ascending alcohol series, and mounted in Eukitt® (O. Kindler). Images were acquired using a Leica TCS SP2 (Leica Microsystems GmbH, Wetzlar, Germany), inverted confocal-laser scanning microscope, Zeiss LSM 880 Airyscan confocal microscope, and a Zeiss AxioZ brightfield microscope (Carl Zeiss Jena, Germany). Images were processed using ImageJ and Adobe Photoshop software. 3D images of Kv2.1^+^ and NRG1^+^ signals were acquired using IMARIS ×64 v.10.0.1 software (Oxford Instruments).

### Cell Counting of Chromogenic Stainings

Immunostainings on 5 μm thick coronal paraffin sections (bregma –1.3 or –1.7) were imaged as tile overviews at 10× magnification using a Zeiss Imager.Z1 microscope. Cell numbers were counted using the Cell Counter plugin for ImageJ software and expressed as cell densities (cells per area counted, in mm^2^) or number of positive cells per region. Dentate gyrus areas were measured using ImageJ software. Cell numbers or areas of both hemispheres were averaged.

### Quantification of GFAP^+^, IBA1^+^, and Kv2.1^+^ Areas

GFAP^+^ and IBA1^+^ areas were quantified on chromogenic immunostainings of coronal brain sections with a semi-automatic analysis using ImageJ software as described.^29^ Overview pictures (10×) were acquired with the Zeiss Imager.Z1 microscope, and ratio of DAB-positive versus total area was quantified in regions of interest (ROI). Area fractions from both hemispheres were averaged. Kv2.1^+^ area fraction and number of Kv2.1^+^ puncta were quantified using fluorescent immunostainings of frozen coronal brain sections. Images were acquired with a 40×/1.0 or 63× DIC objective of a Zeiss LSM 880 Airyscan confocal microscope and examined by semi-automatic analysis using a custom-made macro in FIJI software. The number of Kv2.1^+^ puncta was normalized to the number of DAPI^+^ cells in designated ROI.

### Electrophysiology

Mice (P30) were anesthetized and sacrificed using isoflurane. Brains were removed and 300 μm thick transverse brain sections were prepared from both hemispheres using a Leica VT1200S vibratome in ice-cold sucrose-based Slicing Solution (230 mM Sucrose, 26 mM NaHCO_3_, 2 mM KCl, 1 mM KH_2_PO_4_, 1 mM MgCl_2_, 10 mM Glucose, 0.5 mM CaCl_2_) saturated with carbogen gas (95% oxygen, 5% carbon dioxide). Brain slices were transferred into a holding chamber filled with Artificial Cerebrospinal Fluid (ACSF; 120 mM NaCl, 26 mM NaHCO3, 1 mM KH_2_PO_4_, 2 mM KCl, 2 mM MgCl_2_, 10 mM Glucose, 2 mM CaCl_2_ saturated with carbogen gas) and incubated for at least 20 minutes before recording. An interface recording chamber (BSCBU Base Unit with the BSC-HT Haas Top, Harvard Apparatus) with a constant flow of ACSF at 33°C was used to record **γ**-oscillations in the CA3 region of the hippocampus. Extracellular recording electrodes (with a resistance between 2.0-3.0 MΩ) filled with ACSF solution were placed on the CA3 pyramidal cell layer of slices perfused constantly with the same solution. Baseline recordings were performed before kainate application for 10 minutes. Oscillatory recordings were performed for 20 minutes upon perfusion with 100 nM kainate (in ACSFsolution). In order to find the maximum oscillations, electrodes were then re-positioned and slices were recorded for additional 20 minutes in the presence of kainate. To assess the effect of PD158780 (ErBb receptor inhibitor, MedChemExpress, HY-18609) on **γ**-oscillations, baseline recordings were performed for 20 minutes under ACSF perfusion. Oscillations were induced by 100 nM kainate and recorded for 1 hour to achieve stable maximum **γ**-oscillations. Subsequently, 100 uM of PD158780 was applied in ACSF for 30 minutes, followed by a 20 minutes washout period. Signals were acquired using a Multiclamp 700B amplifier and Digidata 1440A data acquisition system (Axon Instruments, Molecular Devices), with 3 kHz Bessel filter. Data were analyzed using Axograph X software (Axon Instruments). The power spectra were calculated for 10 minutes recording epochs. Baseline power spectra were subtracted from kainate-induced oscillatory spectra, and peak frequency and average power were determined within the 25-45 Hz frequency band.

Voltage–dependent potassium currents were measured in voltage-clamp condition at –70 mV using the HEKA EPC-10 amplifier and Patch-Master (Ver. 2X73.5) software (HEKA electronics). The internal patch pipette solution contained 138 mM K-gluconate, 16.8 mM HEPES, 10 mM NaCl, 1 mM MgCl_2_·6 H_2_O, 0.25 mM EGTA, 4 mM ATP-Mg^2+^, 0.3 mM GTP–Na^+^ (adjusted to pH 7.4, ∼320 mOsmol); the external solution adds TTX (0.3 μM) to ACSF solution. Resistance of recording pipette filled with internal solution was 3–4 MΩ. Membrane current recordings were collected after adjustment of capacitance and series resistance compensation. Serial resistance was compensated by ~50% and only cells with serial resistance below 10 MΩ were analyzed. *I*_K_ currents were evoked by 500 ms depolarizing test pulsed from –80 mV to +70 mV in 10 mV increment after –70 mV. *I*_DR_ was evoked by same voltage steps with a 1000 ms preconditioning pulse of –40 mV to inactivate *I*_A_. *I*_A_ were isolated by digital subtraction of currents between two specific voltage-clamp protocols (*I*_K_-*I*_DR_). Data were analyzed using Axograph (Ver.1.8.0) software (Axograph Scientific). Experiments using Guangxitonxin-1E (GxTX; Alomone Laboratories) were performed to isolate K_v_2.1 currents in CA3 pyramidal neurons. *I*_K_ currents were evoked by 500 ms depolarizing test pulses from –80 mV to +70 mV in 10 mV increment from a holding potential of –70 mV in ACSF containing 0.3 μM TTX in presence or absence of 1 μM GxTX. Kv2.1-mediated current (I_Kv2.1_) were isolated by subtracting the currents recorded in the presence of GxTX from total I_K_ currents.

Potassium current density (pA/pF) was normalized to cell membrane capacitance (pF) from peak outward current amplitude (pA). *I-V* curves were generated by plotting current density values against test potential. Chord conductance of potassium currents was calculated from “*G* = *I*/(*V_m_-V_rev_*)”, where *I* is current amplitude, *V_m_* is test potential, and *V_rev_* is virtual reversal potential. Chord conductance values for each test potential were normalized with maximal chord conductance values (G_max_) and then average normalized conductance values (G/G_max_) were plotted against test potentials fitted with Boltzmann equation built in Prism program.

Spontaneous miniature excitatory/inhibitory postsynaptic currents (mEPSC/mIPSC) were recorded from CA3 pyramidal neurons in mouse hippocampal brain slices during continuous perfusion with ACSF containing 0.3 μM TTX to block AP firing. In whole cell patch clamp configuration, spontaneous mEPSC and mIPSC signals were recorded at a holding potential of –70 mV without any stimulation for 3-5 minutes. To the same slice to examine GxTX effects on spontaneous miniature responses, 1 μM GxTX was additionally applied to the external solution for 10-15 minutes in the same slice. Spontaneous mEPSC or mIPSC events were detected using template-matching algorithm in AxoGraph software (Axograph Scientific).

## Results

Neuronal activity has been implicated in the regulation of NRG1 expression and signaling functions.^21^^,^^30^^,^^31^ In line with a previous study,^21^ we detected NRG1 isoform-specific transcriptional responses upon neuronal stimulation of primary hippocampal and neocortical neurons by KCl treatment. Specifically, we confirmed by qRT-PCR elevated expression of NRG1 type I transcripts, while NRG1 type III expression was not affected, and expression of NRG2, a highly related neuregulin, was reduced under these conditions (Figure 1A; Suppl. Figure 2A, B). Fluorescent in situ hybridization (FISH) demonstrated increased NRG1 expression in a majority of KCl-treated cultured neurons (Suppl. Methods, Suppl. Figure 2C, D), most of which expressed the glutamatergic marker neurogranin (Suppl. Figure 2E). This finding is consistent with FISH analysis of adult brain sections that detected NRG1 type I and type III transcripts in cortical layer VI and the pyramidal cell layer in CA3 (Suppl. Methods, Suppl. Figure 2G, H). These data prompted us to model stage- and glutamatergic neuron-specific NRG1 type I overexpression in vivo, using a conditional transgenic mouse line (*Stop-Nrg1*) for Cre recombinase-mediated expression of N-terminally haemagglutinin (HA) epitope-tagged NRG1 type I^23^ (Figure 1B, C). Based on green fluorescent protein (GFP) expression from a loxP sites-flanked (“floxed”) “Stop” element and immunostaining for neural cell-specific markers, we verified the suitability of this mouse line for Cre-mediated expression in various neural cell types, including glutamatergic neurons (Suppl. Figure 1A, B; Suppl. Figure 3). NRG1 type I expression starts in the embryonic neocortex,^21^^,^^32^ consistent with signaling functions in early brain development. Thus, to model elevated NRG1 type I signaling with an onset during embryonic cortical development, we bred *Stop-Nrg1* mice to *NEX-Cre* driver mice, in which Cre-dependent recombination is initiated around embryonic day (E) 11.5 exclusively in postmitotic glutamatergic projection neurons^24^ (Figure 1C). Double transgenic NEX-Cre^*^Stop-Nrg1 mice (referred to as *NC-Nrg1* mice) were born at expected mendelian frequency and displayed normal lifespan, cage behavior, litter size, and body weight (Suppl. Figure 4A). Immunostaining demonstrated transgenic NRG1 type I expression (and a concomitant loss of GFP) in cortical plate neurons from *NC-Nrg1* mice at E16 (Figure 1D, E). NRG1 type I expression faithfully mimicked *NEXCre* recombination patterns in the hippocampus^24^ (Figure 1F), and co-immunostaining confirmed the absence of NRG1 type I expression from parvalbumin (PV)^+^ GABAergic interneurons in the adult brain (Figure 1G).

**Figure 1:**
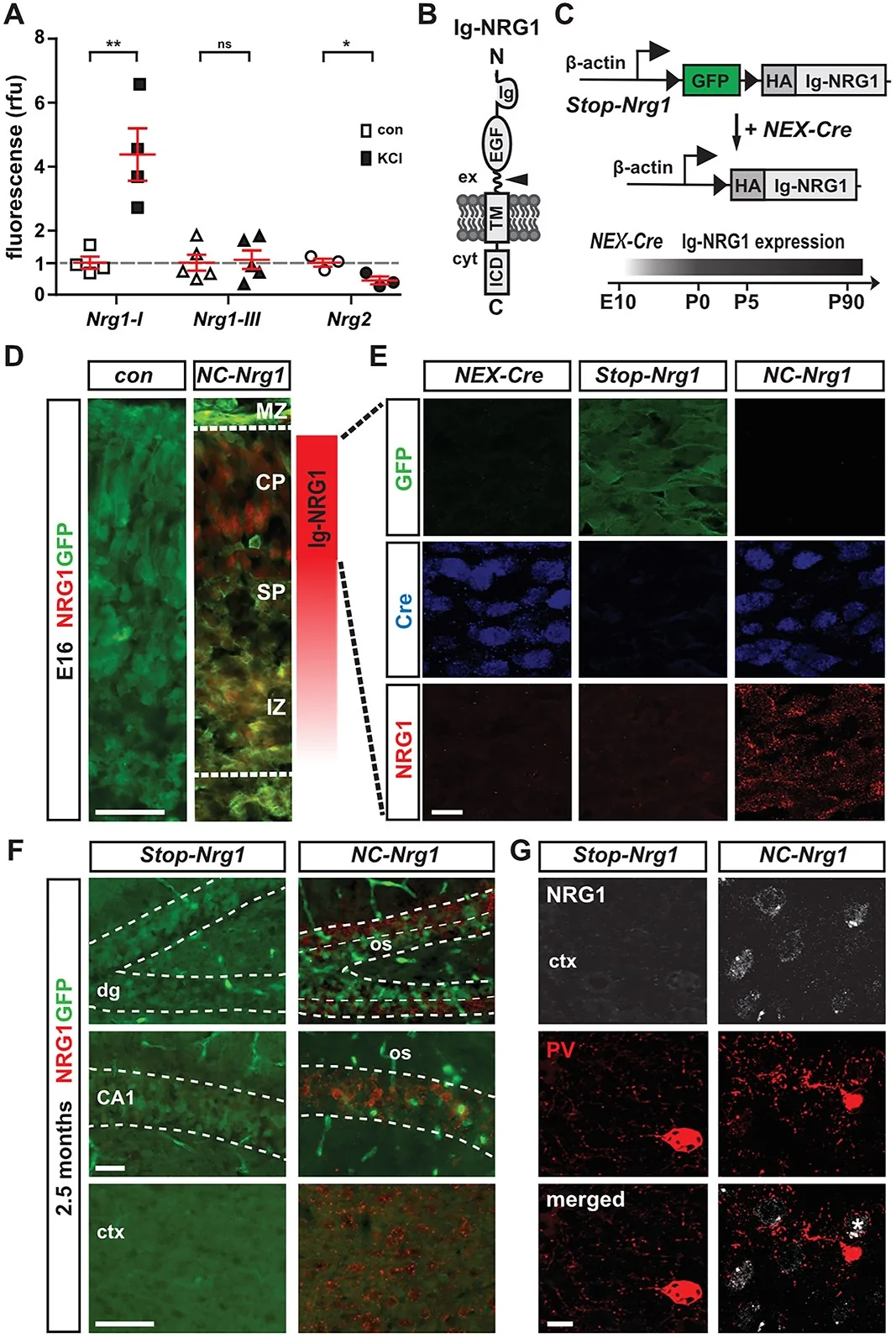
Conditional overexpression of NRG1 Type I in cortical glutamatergic networks. (A) qRT-PCR analysis of primary hippocampal neuron cultures after KCl treatment (50 mM) for 6 hours at 12 DIV. n = 3-4; ^*^P < .05, ^**^P < .01, Ns, *P* > .05; two–tailed T-test. RFU (relative fluorescence units) (B) NRG1 Type I protein domain structure. C, C-terminus; EGF, epidermal growth factor-like; ICD, intracellular domain; Ig, immunoglobulin-like; N, N-terminus; TM, transmembrane domain. Arrowhead, proteolytic cleavage site in the juxtamembrane stalk region. (C) *Stop-Nrg1* mice constitutively express GFP (encoded by a “floxed” stop cassette) under control of the chicken β-actin promoter. Removal of the stop cassette upon breeding to *Cre* driver mice results in expression of HA epitope-tagged NRG1 Type I. *NEX-Cre*-mediated recombination and NRG1 Type I expression (in *NC-Nrg1* mice) Starts at ~E12 in postmitotic cortical projection neurons. Arrowheads, timepoints of analysis in D and H. (D) Transgenic NRG1 Type I expression in the CP and IZ of *NC-Nrg1* embryos at E16. Confocal microscopy of fluorescent immunostaining for GFP and NRG1 (HA Epitope). CP, Cortical plate; IZ, intermediate zone; MZ, marginal zone; SP, subplate; SVZ, subventricular zone. Scale bar, 50 μm. (E) Transgenic NRG1 Type I expression (and concomitant loss of GFP) in CP of *NC-Nrg1* Embryos at E16. Confocal microscopy of fluorescent immunostainings for GFP, Cre, and NRG1 (HA epitope). Scale bar, 10 μm. (F) NRG1 Type I expression in the pyramidal layer of hippocampal CA1 region, granule cells in the outer shell of dentate gyrus, and in neocortex. Fluorescent immunostainings for NRG1 C-Terminus on coronal brain sections. Scale bars, 50 μm; 10 μm (Zoom cx). (G) Neocortical NRG1 Type I expression is absent from PV^+^ Interneurons in adult *NC-Nrg1* mice. Asterisk marks NRG1 Type I-overexpressing glutamatergic neuron contacted by PV^+^ terminals. Confocal images of fluorescent immunostainings on coronal brain sections (age 10 weeks). Scale bar, 15 μm

Biochemical analysis verified *NEXCre*-dependent expression of full-length NRG1 type I in the neocortex of *NC-Nrg1* mice (Figure 2A), and co-immunostaining for N- and C-terminal domains of NRG1 type I (Figure 2B) was consistent with proteolytic processing in the juxtamembrane stalk region.^33^ Next, we compared NGR1 type I expression, ErbB4 receptor activation, and downstream signaling in *NC-Nrg1* mice to a “constitutive” transgenic mouse line with prominent NRG1 type I overexpression in hippocampus and neocortex under control of the neuronal Thy1.2 promoter (*T-Nrg1*^34^; Suppl. Figure 4B). Biochemical analysis of adult brain revealed a ~ 6-fold higher NRG1 type I overexpression in adult *T-Nrg1* mice compared to *NC-Nrg1* mice (Suppl. Figure 4C). To investigate ErbB4 stimulation, we employed an antibody directed against phosphorylated Tyr1284, a well-characterized marker of NRG1–mediated ErbB4 activation by auto-phosphorylation and downstream signaling via adaptor protein Shc,^35^ upstream from both MAP and AKT kinase pathways.^36^ Consistent with higher NRG1 type I overexpression in *T-Nrg1* mice, ErbB4 phosphorylation at Tyr1284 was ~4-fold higher in *T-Nrg1* mice compared to *NC-Nrg1* mice (Figure 2C, D). In line with the embryonic onset of Cre-mediated recombination, NRG1 type I overexpression was detectable in *NC-Nrg1* mice at P7 (Suppl. Figure 4D) and associated with ~1.5-fold ErbB4 hyperphosphorylation (Figure 2E). NRG1 type I overexpression and ErbB4 hyperphosphorylation (~3-fold) were maintained in adult *NC-Nrg1* mice (Suppl. Figure 4E; Figure 2F).

**Figure 2:**
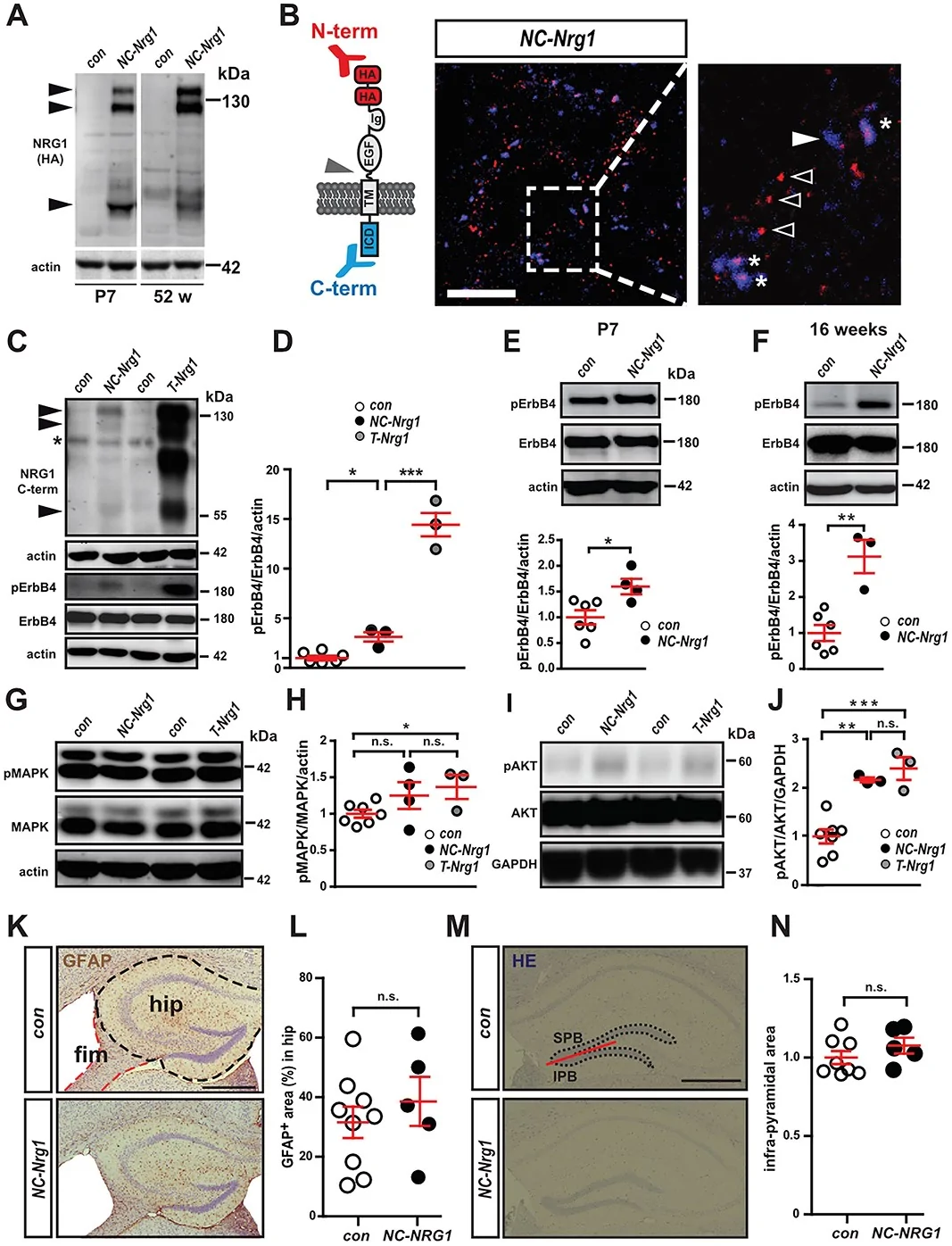
Sustained ErbB4 hyperphosphorylation and AKT kinase stimulation in response to glutamatergic network-specific NRG1 Type I overexpression. (A) Western blotting of PFC Protein lysates from *NC-Nrg1* mice and *Stop-Nrg1* controls at P7 and 52 weeks with antibody directed against the HA epitope. (B) (Left) binding sites for antibodies directed against N- and C-terminal NRG1 Type I domains. Arrowhead, proteolytic cleavage in the juxtamembrane region. (Right) Co-localization (^*^) and Non-overlapping signals for N- and C-terminal domains (empty and filled arrowheads, respectively). Confocal image of fluorescent immunostaining on coronal brain section from a *NC-Nrg1* mouse (Age 6 Weeks). Scale bar, 10 μm. (C) Moderate and marked NRG1 Type I overexpression and concomitant ErbB4 hyperphosphorylation in *NC-Nrg1* and *T-Nrg1* Mice, respectively. Western blotting of PFC protein lysates (Age 16 Weeks) with antibodies directed against the NRG1 C-terminus and pErbB4 (Tyr 1284). Actin was used as loading control. Arrowheads mark two variants of full-length (~130 kDa) and processed NRG1 Type I protein (~50 kDa). Asterisk indicates non-specific band. (D) Densitometric quantification of pErbB4 bands. Integrated density values were normalized to β-actin. n = 3-6 each; ^*^P < .05, ^***^P < .001; T-test (control Vs *NC-Nrg1*; control Vs *T-Nrg1*). (E, F) ErbB4 hyperphosphorylation in the PFC of *NC-Nrg1* mice compared to *Stop-Nrg1* controls at P7 (E) and 16 weeks of age (F). Western blotting using an antibody directed against pErbB4 (tyr 1284). Densitometric quantification of pErbB4 Bands. Integrated density values were normalized to β-Actin. n = 3-6 each; ^*^P < .05, ^**^P < .01; T-test. For corresponding western blot analysis of NRG1 Type I overexpression see Suppl. Figure 4C, D. (G) Western blot analysis of PFC protein lysates (Age 16 Weeks) for MAPK and pMAPK expression. (H) Densitometric quantification of pMAPK bands. Integrated density values were normalized to MAPK and β-Actin loading control; n = 3-4 each; ^*^P = .0222; Ns, *P* > .05; T-test (control Vs *NC-Nrg1*; control Vs *T-Nrg1*). (I) Increased pAKT levels in PFC protein lysates from *NC-Nrg1* and *T-Nrg1* mice compared to *Stop-Nrg1* and *NEX-Cre* controls. (J) Densitometric quantification of pAKT bands as in (H). n = 3-7 each; ^*^P < .05, ^**^P < .01; T-test (control Vs *NC-Nrg1*; control Vs *T-Nrg1*). (K) Chromogenic immunostaining for astrocytes (GFAP) on coronal brain sections from *NC-Nrg1* and control mice (age 16 weeks, bregma -1.3). Quantified areas (Hip, hippocampus; Fim, fimbria) marked by dashed lines. Scale bar, 500 μm. (L) GFAP^+^ Area in the hippocampus of *NC-Nrg1* and control mice (*STOP-Nrg1*, *NEX-Cre*). n = 4-5 each; Ns, *P* > .05; T-test. (M) HE staining of coronal brain sections (Bregma -1.7). Quantified dentate gyrus area marked by dashed line. Line separates supra- and infra-pyramidal regions. Scale bar, 500 μm. (N) No changes in infra-pyramidal dentate gyrus area in *NC-Nrg1* compared to control mice (*STOP-Nrg1*, *NEX-Cre*). n = 4-5 each; Ns, *P* > .05; T-test

Sustained ErbB4 hyperstimulation was linked to increased MAP kinase phosphorylation in *T-Nrg1* mice but not in *NC-Nrg1* mice (Figure 2G, H). In contrast, AKT phosphorylation was significantly increased both in *NC-Nrg1* and *T-Nrg1* mice, with a greater increase observed in *T-Nrg1* mice (Figure 2I, J). These findings imply sustained hyperstimulation of AKT signaling as a possible component of molecular pathophysiology in response to moderate NRG1 type I overexpression.

*T-Nrg1* mice display an elevated hippocampal expression of inflammation-associated markers, including GFAP, and an enlarged dentate gyrus.^37^ In contrast, we found no evidence for astrogliosis or activated microglia in the hippocampus and fimbria of *NC-Nrg1* mice at 16 weeks and even 1 year of age (Figure 2K, L; Suppl. Figure 4F-K). In addition, the number of NeuN^+^ cells in CA1-3 and neocortex (Suppl. Figure 4L, M, O-R), and both infra- (Figure 2M, N) and supra-pyramidal dentate gyrus areas (Suppl. Figure 4N) were not altered in *NC-Nrg1* mice. We conclude that *NC-Nrg1* mice are a physiologically relevant model to examine the effects of moderately increased NRG1 type I signaling activity during embryonic development on juvenile brain functions.

Treatment of hippocampal slices with the recombinant EGF domain of NRG1 increases the power of kainate-induced **γ**-oscillations, proposedly acting via ErbB4 receptors in PV^+^ interneurons.^17^ Adhering to the protocol used for testing the EGF domain,^17^ we induced **γ**-oscillations in hippocampal slices at P30 using 100 nM kainate and performed extracellular field recordings in the stratum pyramidale of CA3 (Figure 3A). In contrast to acute EGF domain treatment, sustained NRG1 type I overexpression in *NC-Nrg1* mice reduced the power of kainate-induced **γ**-oscillations, whereas **γ**-oscillation frequency was not altered (Figure 3B-E; Suppl. Table 1). To rule out a possible effect of *NEX* (*Neurod6*) haploinsufficiency in “knock in” *NEXCre* driver mice, we compared *NEXCre* mice to wildtype, but found no difference in **γ**-oscillation power or frequency (Figure 3F-H; Suppl. Figure 5A-B). Considering the potency of EGF domain treatment in ErbB4 receptor activation in vivo*,*^38^ we additionally tested *NC-Nrg1* mice homozygous for the *Stop-Nrg1* cassette (*NC-Nrg1^hom^;*  Suppl. Figure 5C). However, **γ**-power was also reduced in *NC-Nrg1^hom^* mice (Suppl. Figure 5D-G), suggesting that increasing NRG1 type I signaling levels in vivo is unlikely to switch NRG1 type I functions in hippocampal **γ**-oscillations from inhibitory to stimulatory.

**Figure 3:**
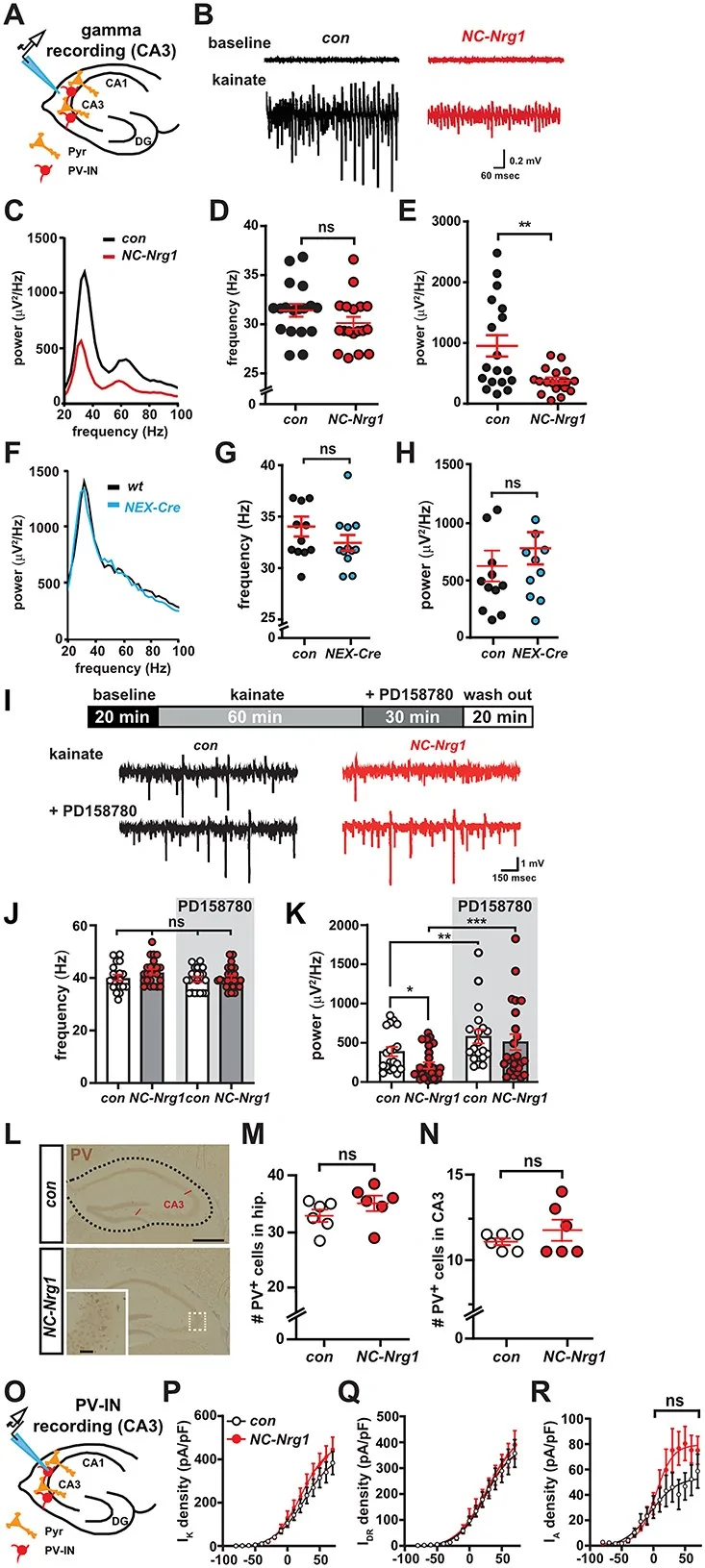
Glutamatergic network-specific NRG1 Type I overexpression reduces the power of kainate-induced **γ**-oscillations in the hippocampal CA3 region. (A) Experimental paradigm for extracellular recordings of kainate-induced **γ**-oscillations in CA3 region. (B) Representative traces of **γ**-Oscillations recorded in CA3 stratum pyramidale before (Baseline) and after kainate application (100 nM) in control (*Stop-Nrg1*) and *NC-Nrg1 Mice* at P30. (C) Representative power spectra of recorded oscillatory activity. (D) the mean **γ**-oscillation frequency at maximum peak of power spectrum was not changed in *NC-Nrg1* mice compared to controls. (E) the average **γ**-oscillation power was reduced in *NC-Nrg1* mice compared to controls. For each recording, the averaged **γ**-oscillation power was analyzed between 25 and 45 Hz frequency in 10 minutes epochs. (n = 18; 3 mice each; Ns, Not significant; ^**^P ≤ .01, student’s T-test). See Suppl. Table 1 for details. (F) Representative power spectrum of **γ**-oscillations in *Wt* and *NEX-Cre* mice. (G, H) No difference in average frequency (G) and average power (H) of **γ**-oscillations (25-45 Hz) between *Wt* and *NEX-Cre* mice. n = 12; 2 mice each (I) Schematic schedule of kainate and PD158780 treatment. Representative **γ**-oscillation traces from control (Left) and NC-Nrg1 mice (Right) before (Top) and during perfusion with 100 μM PD158780 (bottom). Scale bars represent 150 msec and 1 mV. (J) Mean **γ**-oscillation frequency at the maximum peak of power spectrum in control (open symbol) and NC-Nrg1 mice (closed symbol) during PD158780 treatment. (K) Average **γ**-oscillation power in response to PD158780 treatment. PD158780 application increased **γ**-oscillation power in both control and NC-Nrg1 mice. Data are presented as mean ± SEM. Statistical significance was evaluated using student’s T-test; (n = 19-23, 4 mice each; ^*^P < .05; ^**^P < .01; ^***^P < .001). (L) Chromogenic immunostaining for PV on coronal brain sections at p30 (bregma -1.3). Quantified areas are indicated by dashed line (hippocampus) or lines (CA3). Scale bars, 500 μm; 50 μm (magnified images). (M, N) quantification of PV^+^ interneurons in the hippocampus (M) and CA3 region (N) of *NC-Nrg1* mice and controls (*Stop-Nrg1*, *NEX-Cre*). n = 3-6 each; T-test; Ns, not significant. (O) Experimental paradigm for whole-cell patch-clamp recordings in CA3 PV^+^ interneurons. (P–R) current–voltage (*I–V*) relationships of *I*_K_ (P), *I*_DR_ (Q), and *I*_A_ (R) in CA3 PV^+^ Interneurons from control (open symbols) and *NC-Nrg1* mice CA3 PV^+^ interneurons (closed symbols). Data were analyzed using a linear mixed-effects model with cell nested within animal as a random effect, and group and voltage as fixed effects. Data are presented as mean ± SEM. Post hoc comparisons were performed using sidak’s multiple comparisons test (n = 19-21; 5 mice each; ^*^P < .05; ^**^P < .01. ns, not significant)).

To test for a role of ErbB signaling in diminished **γ**-oscillation power in *NC-Nrg1* mice, we repeated recordings in the presence of PD158780, a pan-specific antagonist of ErbB receptor tyrosine kinase activity (Figure 3I). PD158780 treatment had no effect on **γ**-frequency, but increased **γ**-power in controls and restored **γ**-power in *NC-Nrg1* mice to PD158780-treated control levels (Figure 3J, K). As oscillatory activity in the **γ**-band range critically depends on ErbB4-expressing PV^+^ interneuron functions, we next addressed possible changes in PV^+^ interneurons in *NC-Nrg1* mice. Chromogenic immunostainings revealed no differences in the number or localization of these cells in the hippocampus at P30 (Figure 3L-N), as well as hippocampus and neocortex of *NC-Nrg1* mice at 16 weeks and 52 weeks of age (Suppl. Figure 6). Moreover, in contrast to glutamatergic pyramidal cells (see below), we observed no changes in potassium currents, rheobase or AP firing properties in PV^+^ interneurons from *NC-Nrg1* mice compared to controls (Figure 3O-R; Suppl. Figure 5H-L), suggesting unaltered PV^+^ interneuron excitability.

In search for possible glutamatergic neuron-intrinsic deficits that could contribute to diminished **γ**-oscillations under chronic conditions in *NC-Nrg1* mice, we performed immunostainings for NRG1 and a battery of synaptic markers. These studies revealed distinct clusters of NRG1 type I protein in the somatodendritic compartment of glutamatergic neurons from *NC-Nrg1* and *T-Nrg1* mice (Figure 4A, B; Suppl. Figure 7A). Notably, we observed no overlap of NRG1 type I with pre- and postsynaptic compartments of glutamatergic, GABAergic, and serotonergic synapses (Figure 4B; Suppl. Figure 7B-H). We recently showed that NRG1 type I accumulates in the PM of spinal α-motoneurons atop of ER–derived subsurface cisterns.^39^ Thus, we performed co-immunostainings for NRG1 and Kv2.1, a potassium channel that forms micron-sized clusters at homologous ER-PM junctions of cortical neurons.^40^ This revealed a partial overlap of NRG1 type I with Kv2.1-marked ER-PM junctions in *NC-Nrg1* and *T-Nrg1* mice (Figure 4C, D), consistent with previous findings in cultured hippocampal neurons.^41^ To distinguish NRG1 type I accumulation in ER-PM junctions from overexpression-induced “clogging” in the Golgi complex, we carried out co-immunostaining for Golgi marker GM130, but found no evidence for Golgi–associated NRG1 type I expression in *T-Nrg1* mice (Suppl. Figure 7I).

**Figure 4:**
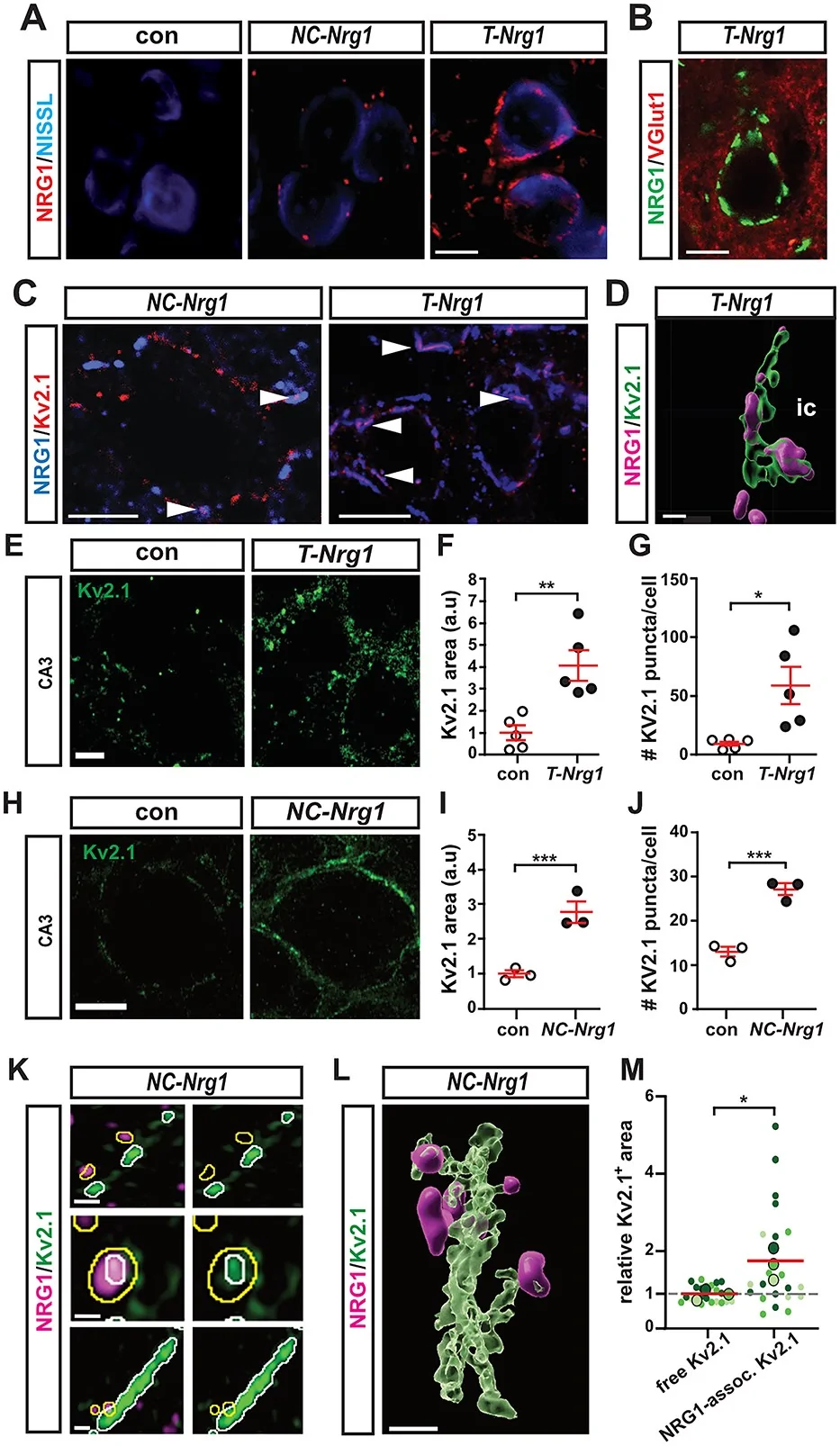
Extra-synaptic accumulation of NRG1 Type I is associated with elevated Kv2.1 clustering in glutamatergic neurons from *NC-Nrg1* and *T-Nrg1* mice. (A) Punctate NRG1 Type I expression domains in the somatodendritic compartment of neocortical neurons (Layer V). Fluorescent immunostainings on coronal brain sections from control, *NC-Nrg1* and *T-Nrg1* mice (Age 8 Weeks) for the C-terminal domain of NRG1. Scale bar, 10 μm. (B) NRG1 Type I puncta in neocortical layer v neuron are non-overlapping with presynaptic glutamatergic marker VGlut1. Fluorescent co-immunostaining on coronal brain section from *T-Nrg1* mouse. Scalebar, 10 μm. (C) Partial colocalization of the c-terminal domain of NRG1 Type I with Kv2.1 in *NC-Nrg1* mice, which is more pronounced in *T-Nrg1* mice. Confocal images of fluorescent immunostainings on coronal brain sections (Age 6 Weeks). Scale bar, 5 μm (Left), 10 μm (right). (D) 3D reconstruction of separated surfaces (Imaris) for Kv2.1 and NRG1 signals in hippocampal pyramidal neuron from a *T-Nrg1* mouse (age 10 Weeks). Ic, intracellular. Scalebar, 0.7 μm. (E) Fluorescent immunostainings for Kv2.1 on coronal brain sections from wildtype and *T-Nrg1* mice (age 10 weeks). Scalebar, 5 μm (40× confocal images). (F) Kv2.1^+^ area fraction and (G) Number of Kv2.1^+^ puncta/CA3 pyramidal neuron. (H) Fluorescent immunostaining for Kv2.1^+^ on coronal brain sections from control and *NC-Nrg1* mice (Age 12 Weeks). Scale bar, 5 μm (63× confocal images). (I) Kv2.1^+^ Area fraction and (J) Number of Kv2.1^+^ puncta/CA3 pyramidal neuron in CA3. n = 3 mice each; *P* < .001; T-test. (K) Fluorescent co-immunostaining for Kv2.1 and HA epitope reveals clustering patterns of Kv2.1 relative to HA-Aggregates in CA3 Pyramidal neurons of NC-Nrg1 mice. Scale bar, 1 μm. (L) Representative 3D surface reconstruction of a Kv2.1^+^ cluster (green) in association with HA-Tagged NRG1 (magenta), acquired using imaris ×64 v.10.0.1 (oxford instruments). Scale bar, 2 μm. (M) Quantification of HA-associated Kv2.1^+^ clusters revealed a significant increase of relative cluster area compared with non-associated (“Free”) Kv2.1^+^ patches in CA3 pyramidal neurons of *NC-Nrg1* mice. Analysis was performed using ZEISS ZEN intellesis threshold segmentation. N = 3 mice each; *P* < .05; T-test

Next, we investigated if increased extra-synaptic NRG1 type I accumulation in transgenic mice has an impact on ER-PM junctions. Immunostaining revealed that CA1 and CA3 pyramidal neurons in *T-Nrg1* mice displayed an increase in the number and size of Kv2.1 clusters compared to wildtype (Figure 4E-G; Suppl. Figure 7J, K), without affecting pyramidal cell density in CA3 (Suppl. Figure 7L). A corresponding analysis confirmed that also a lower level of NRG1 type I overexpression in *NC-Nrg1* mice increases Kv2.1 cluster number and size in CA3 pyramidal neurons (Figure 4H-J), without affecting CA3 pyramidal cell density (Suppl. Figure 7M). Examination of individual CA3 pyramidal cells in *NC-Nrg1* mice confirmed an increased average size of Kv2.1 clusters that overlap with NRG1 type I compared to Kv2.1 clusters that are non-overlapping with NRG1 type I (Figure 4K-M). We also noted a large variability in size for NRG1 type I-overlapping Kv2.1 clusters (Figure 4M), consistent with the notion that NRG1 type I may serve “seeding” functions to promote dynamic changes in Kv2.1-marked ER-PM junctions.

The voltage–gated potassium channel Kv2.1 produces delayed rectifying potassium currents, but also serves non-conducting functions in ER-PM junctions^40^ that may impact on hippocampal network–relevant pyramidal cell properties. Electrophysiological recordings in CA3 pyramidal neurons revealed that total potassium current (I_K_) was reduced in *NC-Nrg1* mice at depolarized potentials, whereas delayed rectifying current (I_DR_) was unaffected (Figure 5C). In contrast, A-type current (I_A_), isolated by voltage-step subtraction, was significantly reduced at depolarizing voltages (Figure 5A-D). Notably, the conductance–voltage (G/G_max_) curves were superimposable between *NC-Nrg1* mice and controls (Suppl. Figure 8B), indicating preserved voltage dependence of channel activation and inactivation, and that I_A_ reduction likely reflects altered channel expression or membrane trafficking rather than changes in gating properties. Pyramidal cell capacitance was equivalent between control and *NC-Nrg1* mice (Suppl. Figure 8C), ruling out differences in somatic size as a contributor to reduced current density. To test a role of Kv2.1-mediated currents in diminished I_A_, we applied the Kv2–specific blocker Guangxitoxin-1E (GxTX) to hippocampal slices. Baseline recordings in response to depolarizing voltage-step pulses before GxTX treatment confirmed a reduction of total I_K_ in CA3 pyramidal neurons from *NC-Nrg1* mice. Following GxTX application, recordings from the same pyramidal cells showed a similar reduction of I_K + GxTX_ currents in controls and *NC-Nrg1* mice, ie, GxTX treatment had no ameliorating effect on I_A_ in *NC-Nrg1* mice (Suppl. Figure 8D). These findings indicate that Kv2.1-mediated delayed rectifying currents have no major role in diminishing I_A_ in pyramidal cells of *NC-Nrg1* mice.

**Figure 5:**
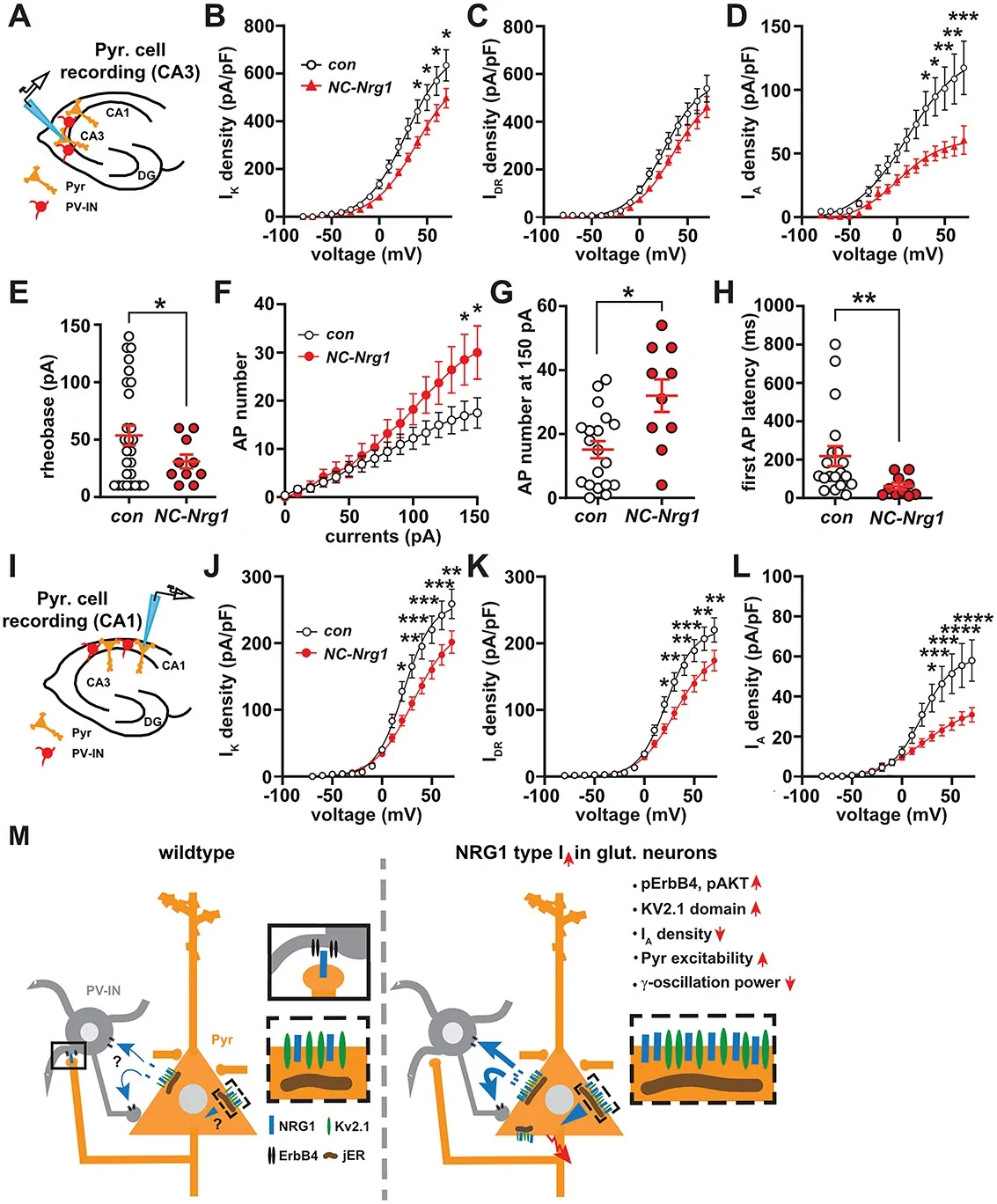
Reduced I_A_ potassium currents and increased excitability in pyramidal neurons from *NC-Nrg1* mice (A) Experimental paradigm for whole-cell patch-clamp recordings from CA3 pyramidal neurons. (B–D) Current–voltage (*I–V*) relationships of *I*_K_ (B), *I*_DR_ (C), and *I*_A_ (D) in CA3 pyramidal neurons (n = 12-17; 4 mice each). (E) Scatter plot of rheobase current (pA) in CA3 pyramidal neurons (n = 10-27). (F) Number of APs as a function of injected current in CA3 pyramidal neurons (n = 10-19). (G) Scatter plot of AP number evoked by a current injection of 150 pA in CA3 pyramidal neurons (n = 10-19). (H) Scatter plot of the latency to first AP (Ms) in response to rheobase current injection in CA3 pyramidal neurons (n = 10-19). (I) Experimental paradigm for whole-cell patch-clamp recordings from CA1 pyramidal neurons. (J–L) Current–voltage (*I–V*) relationships of *I*_K_ (J), *I*_DR_ (K), and *I*_A_ (L) in CA1 pyramidal neurons (n = 21-24). All *I–V* relationships and AP current-response curve were analyzed using a linear mixed-effects model with cell nested within animal as a random effect and group and voltage as fixed effects. All data are presented as mean ± SEM. Post hoc comparisons were performed using sidak’s multiple comparisons test (^*^P < .05; ^**^P < .01; ^***^P < .001; ^****^P < .0001). (M) Working model. (Left) NRG1 Type I is not detected in presynaptic glutamatergic terminals (Beige), proximal to postsynaptic ErbB4 receptors in PV^+^ interneurons (Grey; Black Box). Instead, extra-synaptic NRG1 Type I colocalizes with Kv2.1 in jER (dashed box), consistent with jER-Linked NRG1 Type I functions (Blue Arrowhead). In addition, proteolytic “Shedding” produces “Soluble” NRG1 Type I that signals to ErbB4 in PV^+^ interneurons (blue arrows). (Right) elevated paracrine NRG1 Type I signaling in transgenic mice hyperstimulates ErbB4 receptors and downstream signaling pathways in PV^+^ interneurons. Increased NRG1 Type I protein accumulates at jER, promotes jER remodeling (dashed box), Reduces a-type potassium channel currents, increases pyramidal neuron excitability, and disturbs **γ**-oscillation activity. PV-IN, parvalbumin-expressing interneuron; Pyr, pyramidal neuron; jER, junctional ER

Because I_A_ sets action potential (AP) threshold and limits repetitive firing, we next examined whether its reduction translates into changes in pyramidal neuron excitability. Current-clamp recordings from CA3 pyramidal neurons revealed a lower rheobase, an increased number of evoked APs, a greater AP number at 150 pA current injection, and a shorter latency to the first AP (Figure 5E-H). These findings were further supported by representative recordings from CA3 pyramidal neurons, which showed more robust AP discharge at rheobase and suprathreshold stimuli in *NC-Nrg1* mice (Suppl. Figure 8E). A corresponding analysis in CA1 pyramidal cells demonstrated a reduction in total, A-type, and even delayed rectifier potassium currents (Figure 5I-L), demonstrating that suppression of potassium currents, particularly I_A_, is a common feature of hippocampal pyramidal neurons, and consistent with a general increase in pyramidal cell excitability and altered spike timing coherence in the hippocampus of *NC-Nrg1* mice.

In summary, we found no evidence for NRG1 type I expression in presynaptic glutamatergic terminals, even under conditions of prominent overexpression in transgenic mice. Instead, we suggest a working model (Figure 5M), according to which NRG1 type I accumulates in the somatodendritic compartment of glutamatergic neurons outside postsynaptic sites, with a substantial fraction of NRG1 type I located to ER-PM junctions. Chronically elevated NRG1 type I expression in glutamatergic neurons impairs hippocampal **γ**-oscillations, as a consequence of ErbB4 hyperstimulation in PV^+^ interneurons and maladaptive remodeling in glutamatergic networks, including altered ER-PM junctions, decreased A-type potassium currents, hyperexcitability, and reduced spike timing coherence (Figure 5M).

## Discussion

Insight into pathological processes linked to individual genetic risk factors of schizophrenia is limited and requires the interrogation of adequate mouse models. We provide evidence that moderate NRG1 type I overexpression in *NC-Nrg1* mice causes persistent ErbB4 receptor hyperstimulation in the brain without affecting hippocampal morphology or inducing neuroinflammation, in stark contrast to “conventional” *T-Nrg1* mice.^37^ We conclude that *NC-Nrg1* mice are a physiologically more relevant model to examine how developmental hyperactivity of a “genetic risk pathway” translates into neuronal network dysfunctions with relevance for neuropsychiatric disorders.

Our electrophysiological examination of *NC-Nrg1* mice revealed a reduction in the power of kainate-induced **γ**-oscillations in the hippocampal CA3 region. This suggests abnormal oscillatory network activity in adolescent *NC-Nrg1* mice as a consequence of developmental hyperactivity of NRG1 type I signaling and matches increased **γ**-power in mouse mutants with ErbB4 disruption in PV^+^ interneurons.^18^ However, it contrasts previous studies, in which acute treatment with the soluble EGF domain of NRG1 (NRG1β increased kainate-induced **γ**-power in CA3).^17^ These discrepancies suggest differences in mechanism of action between acute exposure to extremely potent signaling activity of recombinant NRG1β versus chronic signaling of full-length NRG1 type I to ErbB4 receptors, which may induce long-term homeostatic and maladaptive changes in hippocampal networks. Of note, *T-Nrg1* mice display a reduced frequency, but normal power of carbachol-induced **γ**-oscillations in CA3,^42^ pointing to distinct network dysfunctions in response to significantly higher NRG1 type I expression in *T-Nrg1* mice.

What are potential pathomechanisms for reduced **γ**-oscillation power in *NC-Nrg1* mice? NRG1-mediated ErbB4 signaling in PV^+^ interneurons regulates the development of GABAergic circuitry^43^ and cortically expressed NRG1 type I serves as an attractant for cortically migrating PV/ErbB4^+^ interneurons,^32^ suggesting that embryonic NRG1 type I/ErbB4 hyperstimulation could impair PV^+^ interneuron development in *NC-Nrg1* mice. We observed unaltered numbers and location of PV^+^ interneurons in the hippocampus and neocortex of *NC-Nrg1* mice. However, glutamatergic neuron-specific overexpression of cysteine-rich domain-containing NRG1 diminishes inhibitory functions^44^ and PV^+^ interneurons from *T-Nrg1* mice exhibit reduced NMDA receptor-mediated currents.^45^ Thus, more subtle developmental changes in PV^+^ interneuron functions may add to impaired **γ**-oscillations in *NC-Nrg1* mice. Importantly, we show that acute pharmacological blockade with a panErbB inhibitor fully recovered **γ**-power in hippocampal slices from *NC-Nrg1* mice, in line with enhanced oscillatory activity after ErbB4 deletion in PV^+^ interneurons.^18^ This indicates that NRG1 type I/ErbB4 signaling mediates “constraining” functions that counteract **γ**-oscillatory activity, eg, via regulation of PV^+^ interneuron excitability through potassium channel Kv1.1.^46^ However, as we found no evidence for altered PV^+^ interneuron excitability or inhibitory drive on pyramidal cells in *NC-Nrg1* mice, PV^+^ interneuron-intrinsic (patho)mechanisms downstream from NRG1 type I/ErbB4 hyperstimulation require further investigation.

Optogenetic interference with pyramidal cell excitability reduces hippocampal **γ**-power,^47^ in line with pyramidal cell-intrinsic changes in excitability that could contribute to altered **γ**-oscillations under chronic conditions in *NC-Nrg1* mice. This notion is supported by a glutamatergic hypofunction in mice with CamKII/tTA-dependent postnatal NRG1 type I overexpression, which was reported to result from pyramidal cell-intrinsic, ErbB4-independent processes.^48^ Accordingly, we found no evidence for NRG1 type I expression in presynaptic glutamatergic terminals, calling into question “point-to-point” presynaptic signaling to postsynaptic ErbB4 receptors in PV^+^ interneurons as a major driver of abnormal **γ**-oscillations under chronic conditions. Instead, we demonstrate extra-synaptic NRG1 type I accumulation at Kv2.1-marked ER-PM junctions, which is congruent with findings in NRG1 type I-transfected hippocampal neurons. ^41^ Similar to effects of NRG2 overexpression in cultured ErbB4^+^ interneurons,^49^^,^^50^ we found that elevated NRG1 type I expression in *NC-Nrg1* mice was associated with an increase in number, size, and possibly structural dynamics of somatic Kv2.1 puncta. In line with biochemical studies on NRG2,^50^ we speculate that NRG1 type I and Kv2.1 share the same tethering mechanisms at ER-PM junctions via molecular interactions with the ER scaffolding protein VAP (VAMP-associated protein), thereby tuning their activity in response to changes in excitatory activity. Importantly, aggregated KV2.1 channels most likely serve non-conducting functions.^51^ Thus, an increased number or size of Kv2.1 puncta in *NC-Nrg1* mice is not necessarily linked to elevated Kv2.1-mediated currents. Rather, we suggest that aggregated NRG1 type I and Kv2.1 channels organize ER-PM junctions, including clustering and functional coupling of voltage-gated L-type Ca^2+^ channels in the PM with ER-located ryanodine receptors, thereby linking neuronal activity with local Ca^2+^ signaling.^52–54^ Moreover, ryanodine receptor activation has been associated with PKA-mediated downregulation of A-type potassium channel currents in hippocampal neurons,^55^^,^^56^ providing a possible mechanism for reduced A-type potassium currents in pyramidal neurons from *NC-Nrg1* mice. Ultimately, diminished A-type potassium currents may result in pyramidal cell hyperexcitability and loss of coherent spike timing, with relevance for the expression of **γ**-oscillatory activity.

We recently reported an altered structure of specialized ER-PM junctions in spinal cord motor neurons and their associated cholinergic C-boutons in NRG1 type I and type III transgenic mice,^39^^,^^57^ consistent with a role for NRG1/Kv2.1 interactions at ER-PM junctions in the regulation of α-motorneuron excitability. Finally, we note that NRG1 type I overexpression using NEX-Cre driver mice occurs also outside the hippocampus, mainly in glutamatergic neurons of the neocortex, and in a smaller number of mid- and hindbrain neurons.^24^ Based on our observations of extra-synaptic NRG1 type I accumulation in neocortical glutamatergic neurons, we speculate that altered excitability and abnormal **γ**-power may also a feature in the neocortex of NC-Nrg1mice.

Taken together, we propose a working model in which NRG1 type I accumulation in pyramidal cells causes sustained ER-PM junction remodeling, including an increased (indirect) Kv2.1 recruitment, which in turn diminishes A-type potassium channel functions. NRG1 type I accumulation may also affect other ER-PM junction-associated ion channels with important functions in neuronal excitability and **γ**-oscillations, eg, calcium-gated (SK) potassium channels.^58^ Ultimately, this could interfere with optimal reciprocal coupling between pyramidal cells and PV^+^ basket cells, adding to impaired **γ**-oscillation power. Exactly how NRG1 type I accumulation alters ER-PM junction remodeling is currently unclear. Formally, ErbB4 hyperphosphorylation in *NC-Nrg1* mice implies paracrine NRG1 type I signaling to PV^+^ interneuron ErbB4 receptors.^41^ However, a recent study reported a reduction of Nav1.1 sodium channel currents in transgenic mice with NRG1 type I overexpression in GABAergic interneurons in the absence of elevated ErbB4 stimulation.^59^ Thus, extra-synaptic NRG1 type I accumulation at ER-PM junctions may primarily act by providing a platform for (abnormal) protein/protein interactions and/or nuclear back signaling via its cytoplasmic domain.^60^

A remarkable convergence of abnormal NRG1/ErbB4 expression, PV^+^ interneuron deficits, and disordered oscillatory network activity has been noted in several neurological disorders, including schizophrenia, multiple sclerosis, epilepsy, and Alzheimer’s disease.^61–64^ We propose the concept of a NRG1-mediated “signalopathy” as a common pathophysiological outcome, in which (NRG1-dependent and -independent) PV^+^ interneuron deficits drive neuronal hyperexcitability. This could result in a vicious cycle of NRG1 accumulation at ER-PM junctions, possibly amplified by human *NRG1* gene variants prone to increased *Nrg1* transcription,^65^ and, ultimately, abnormal **γ**-oscillations. Aberrant oscillatory network activity may serve as a sensitive biomarker during prodromal neuropsychiatric disease stages that could be read out by non-invasive EEG recordings.^66^ Thus, pathomechanistic insight into NRG1-mediated neuronal network dysfunctions will contribute to better diagnosis and treatment strategies in neuropsychiatric disorders.

## Supplementary Material

Supplementary_files_sbag168

## Statistics

Data obtained from biochemical, immunohistochemical, and electrophysiological analyses were analyzed with GraphPad Prism 9.0 software. One-way ANOVA analyses were performed when more than one group of controls were included in the experiment. Bonferroni-post hoc tests for pairwise group comparisons were performed. If no differences between control groups were observed, these were pooled and t-tests were performed. Data are displayed as means ± SEM.
