# Supplementary material for: Extra-Synaptic Functions of NRG1 Type I Signaling in Hippocampal γ-Oscillations
Source: Schizophr Bull. 2026 Oct 30;52(6):sbag168. doi: 10.1093/schbul/sbag168 (PMC13720722; doi:10.1093/schbul/sbag168)
Supplement: Supplementary file 1 [file supplementary_files_sbag168.zip › Supplementary_Figure_1_sbag168.pdf]

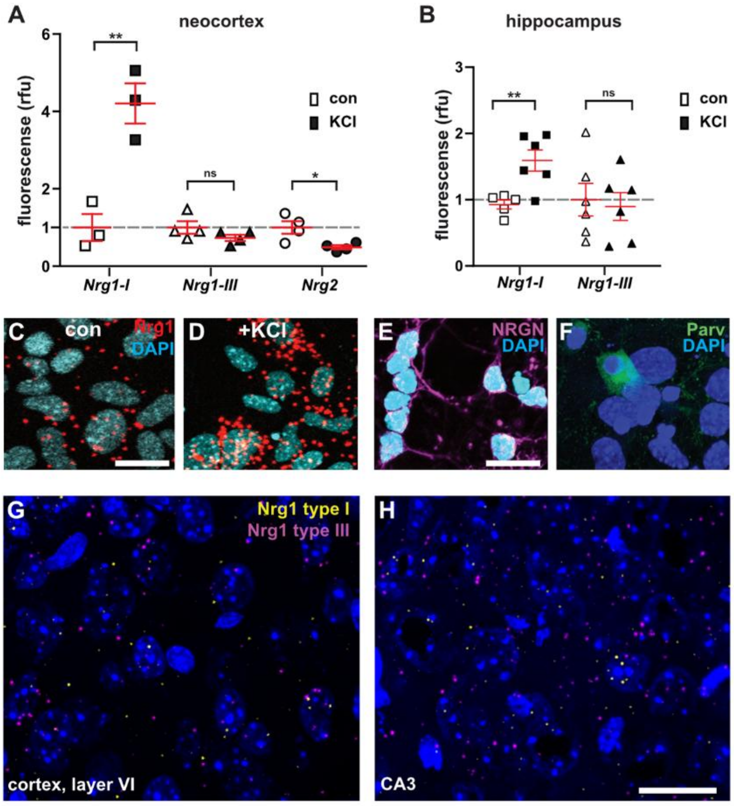

Suppl. Fig. 1. Neuronal activity-dependent induction and endogenous expression of Nrg1 mRNA in mouse CNS neurons.

(A) qRT-PCR analysis of primary neocortical neuron cultures after KCl treatment (50 mM) for 6 h at 12 DIV. n = 3-4; *p<0.05, **p<0.01, ns, p>0.05; two tailed t-test. rfu, (relative fluorescence units). (B) qRT-PCR analysis of primary hippocampal neuron cultures following KCl treatment (50 mM, 6 h) at 12 DIV, confirming the findings shown in Figure 1. $n = 4–6$; *$p < 0.01$; ns, $p > 0.05$; two-tailed t-test. rfu, (relative fluorescence units). (C, D) RNAscope™ fluorescent *in situ* hybridization images from primary hippocampal neuron cultures at 12 DIV following KCl treatment (50 mM, 6 h) showing increased Nrg1 mRNA expression in (D) KCl-treated cells compared with (C) untreated controls. Scale bar, 20 µm. (E, F) Fluorescent immunostaining for Neurogranin (NRGN; E) and Parvalbumin (Parv; F) demonstrate that the majority of cultured neurons are excitatory projection neurons, whereas only a small number of interneurons are present. Nuclei were counterstained with DAPI (blue). Scale bar, 20 µm. (G, H) RNAscope™ fluorescent *in situ* hybridization images of fresh-frozen coronal brain sections from 8-week-old WT mice processed using the RNAscope™ Multiplex Fluorescent Reagent Kit v2. NRG1 Type I mRNA is shown in yellow, NRG1 Type III mRNA in magenta, and nuclei were counterstained with DAPI (blue). (G) Cortical layer VI, (H) hippocampal CA3 region. Scale bars, 20 µm.
