# Supplementary material for: Extra-Synaptic Functions of NRG1 Type I Signaling in Hippocampal γ-Oscillations
Source: Schizophr Bull. 2026 Oct 30;52(6):sbag168. doi: 10.1093/schbul/sbag168 (PMC13720722; doi:10.1093/schbul/sbag168)
Supplement: Supplementary file 1 [file supplementary_files_sbag168.zip › Supplementary_Figure_2_sbag168.pdf]

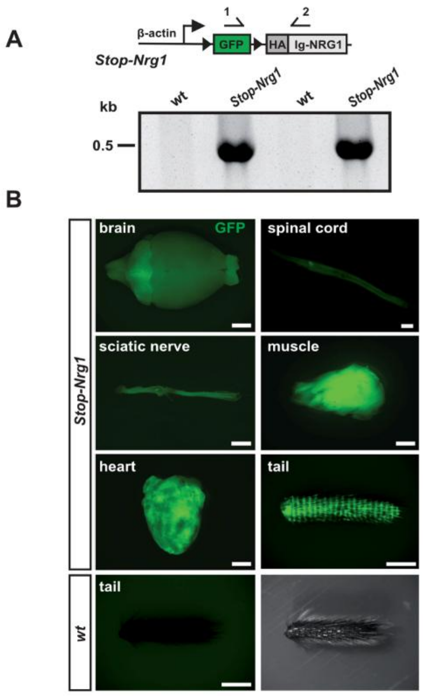

Suppl. Fig. 2: *Stop-Nrg1* transgene expression in nervous and non-nervous tissues.

(A) *Stop-Nrg1* transgene cassette. PCR on genomic tail DNA (location of primers 1, 2 indicated above) shows genomic integration of the transgene cassette in *Stop-Nrg1* mice. (B) In the absence of Cre recombinase *Stop-Nrg1* mice display green fluorescence in various tissues due to GFP expression from the floxed GFP (Stop) cassette.
