# Supplementary material for: Extra-Synaptic Functions of NRG1 Type I Signaling in Hippocampal γ-Oscillations
Source: Schizophr Bull. 2026 Oct 30;52(6):sbag168. doi: 10.1093/schbul/sbag168 (PMC13720722; doi:10.1093/schbul/sbag168)
Supplement: Supplementary file 1 [file supplementary_files_sbag168.zip › Supplementary_Figure_3_sbag168.pdf]

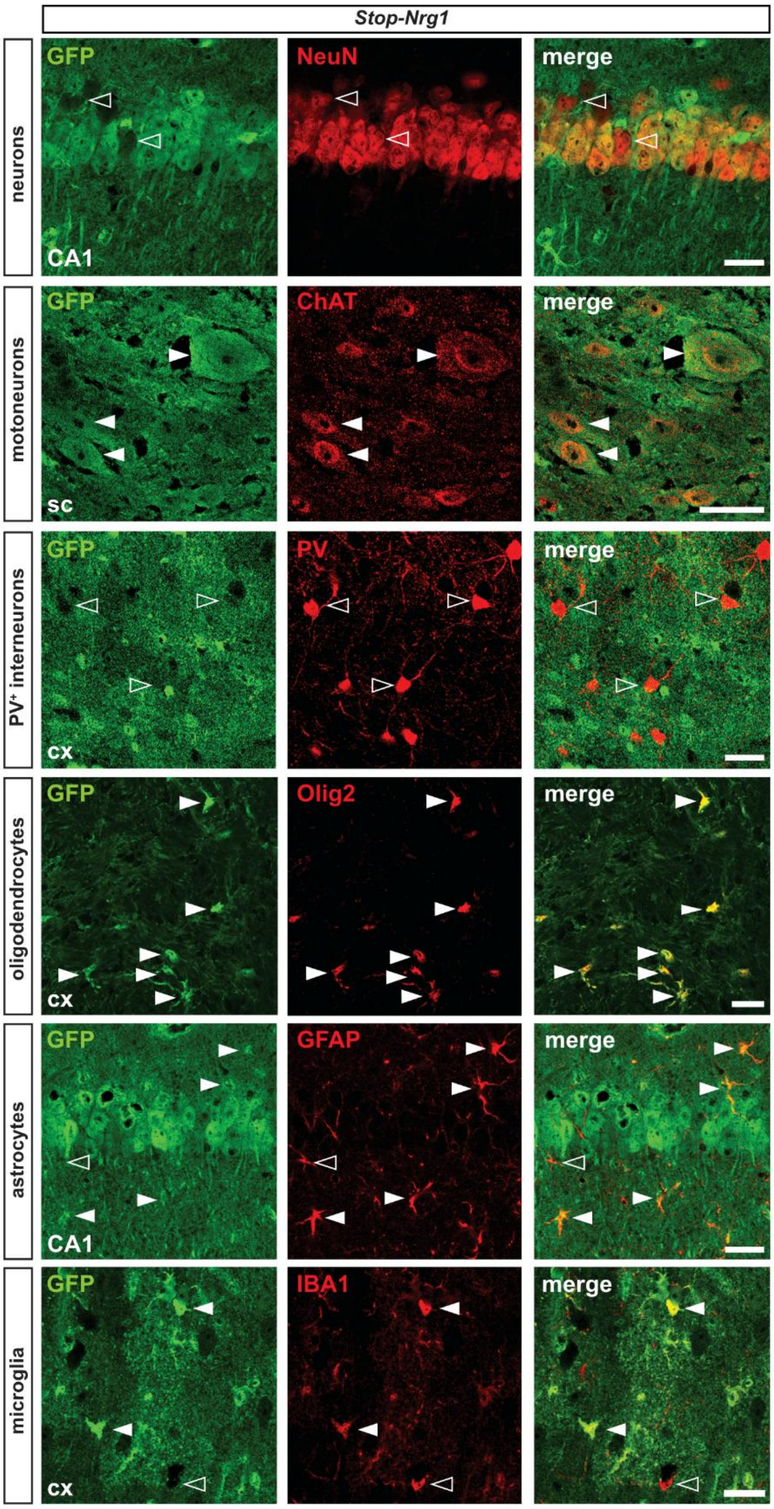
Stop-Nrg1
neurons
GFP
NeuN
merge
CA1
motoneurons
GFP
ChAT
merge
sc
PV+ interneurons
GFP
PV
merge
cx
oligodendrocytes
GFP
Olig2
merge
cx
astrocytes
GFP
GFAP
merge
CA1
microglia
GFP
IBA1
merge
cx

Suppl. Fig. 3: *Stop-Nrg1* transgene expression in neural cell types.

The *Stop-Nrg1* transgene is expressed in hippocampal pyramidal neurons (NeuN) and spinal cord α-motor neurons (ChAT), but is not suitable for expression in GABAergic PV^+ cortical interneurons. Immunostainings for glial markers reveals transgene expression in oligodendrocytes (Olig2) and a subset of astrocytes (GFAP) and microglia (IBA1). Filled arrowheads point to GFP-positive cells, empty arrowheads point to GFP-negative cells. Confocal images of fluorescent immunostainings on coronal brain sections (bregma -1.7) from *Stop-Nrg1* mice (age 3 months). Scale bars, 20 µm.
