# Supplementary material for: Extra-Synaptic Functions of NRG1 Type I Signaling in Hippocampal γ-Oscillations
Source: Schizophr Bull. 2026 Oct 30;52(6):sbag168. doi: 10.1093/schbul/sbag168 (PMC13720722; doi:10.1093/schbul/sbag168)
Supplement: Supplementary file 1 [file supplementary_files_sbag168.zip › Supplementary_Figure_4_sbag168.pdf]

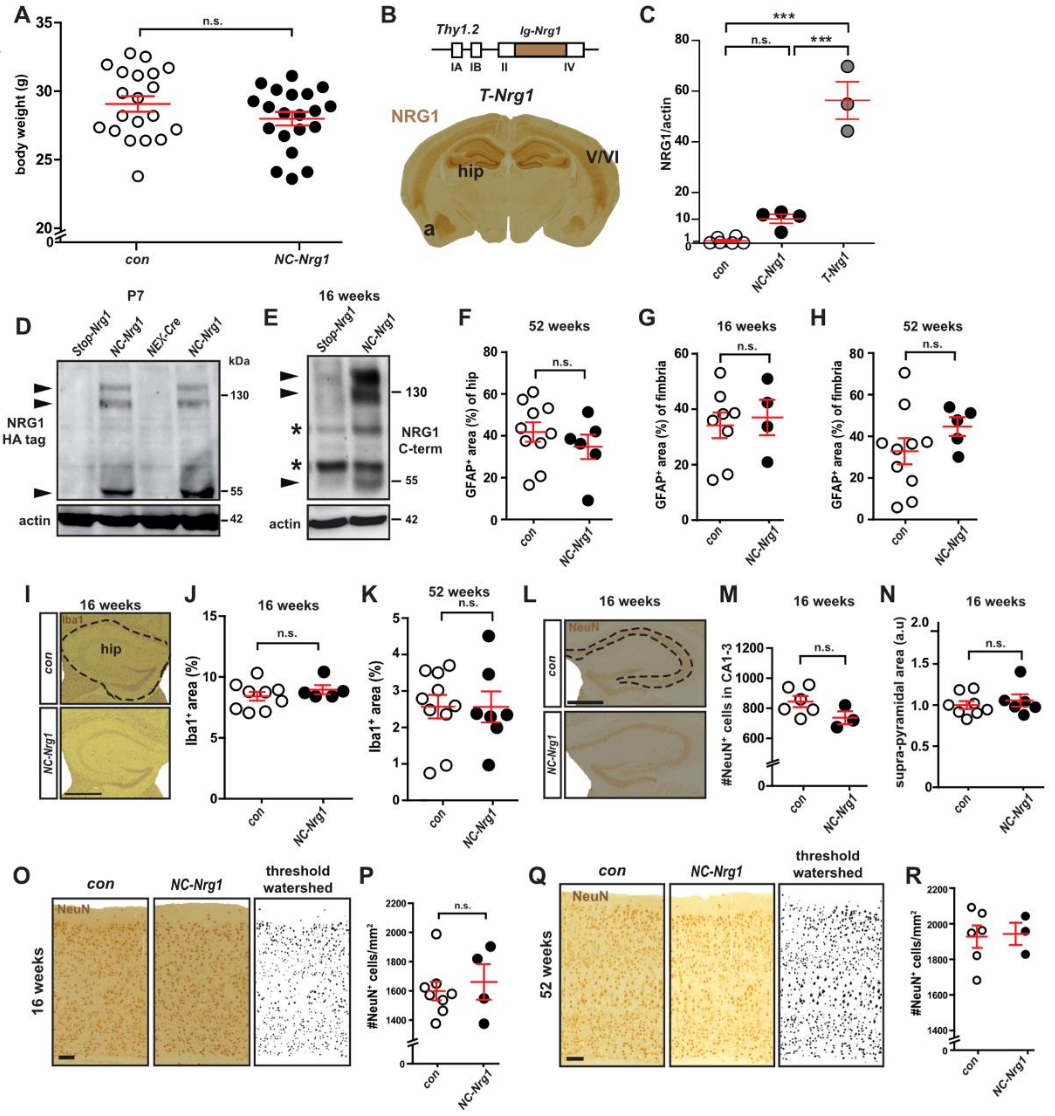

Suppl. Fig. 4: No signs of neuroinflammation and -degeneration in *NC-Nrg1* mice.

(A) Unaltered body weight in male *NC-Nrg1* mice compared to controls (*Stop-Nrg1*, *NEX-Cre*) at 16 weeks of age. (B) Pronounced NRG1 type I expression under control of the neuronal Thy1.2 promoter (see cartoon of transgene cassette; Michailov et al., 2004) in the brain (including hippocampus) of *T-Nrg1* mice (age 8 weeks). Chromogenic immunostaining of coronal brain section from a *T-Nrg1* mouse labelling the C-terminal domain of NRG1. a, amygdala; hip, hippocampus; V/VI, neocortical layers. (C) Adult brains (age 16 weeks) of *T-Nrg1* mice display a ~6-fold NRG1 type I overexpression compared to *NC-Nrg1* mice, which show a ~10-fold NRG1 type I overexpression compared to controls (*Stop-Nrg1*, *NEX-Cre*). Note that ~10-fold increase results from very low NRG1 expression in control brains. For corresponding western blot of PFC protein lysates see Fig. 2C. Densitometric quantification of full-length NRG1. Integrated density values were normalized to β-actin. n=3-6 each; ***p<0.001; t-test. (D) Transgene-derived NRG1 type I expression in *NC-Nrg1* mice at P7. Western blotting of PFC protein lysates (also used to determine ErbB4 phosphorylation in Fig. 2E) with antibody directed against HA epitope. (E) NRG1 type I overexpression in the brain of *NC-Nrg1* mice (age 16 weeks). Western blot

analysis of PFC lysates (also used to determine ErbB4 phosphorylation in Fig. 2F) using an antibody directed against the NRG1 C-terminal domain. (F) Quantification of GFAP$^+$ area in the hippocampus following chromogenic immunostaining of coronal brain sections (bregma -1.3, age 52 weeks). For quantified area see Fig. 2K. n=5-6 each; t-test, ns, not significant. (G, H) Quantification of GFAP$^+$ area in hippocampal fimbria at 16 weeks (G) and 52 weeks of age (H). For quantified area see Fig. 2K. n=4-10 each; t-test, ns, not significant. (I) Chromogenic immunostainings of coronal brain sections (bregma -1.3) for Iba1 (age 16 weeks). Quantified area marked by dashed line. (J, K) Quantification of Iba1$^+$ covered area in the hippocampus of *NC-Nrg1* mice and controls (*Stop-Nrg1*, *NEX-Cre*) at 16 weeks (I) and 52 weeks of age (J). n=5-9 each; t-test, ns, not significant. (L) Chromogenic immunostainings of coronal brain sections (bregma -1.3) for NeuN (age 16 weeks). Quantified CA1-3 area marked by dashed line. (M) Quantification of NeuN$^+$ neurons. n=3-6 each; t-test, ns, not significant. (N) Unaltered supra-pyramidal dentate gyrus area in *NC-Nrg1* compared to controls (*Stop-Nrg1*, *NEX-Cre*; see Fig. 2M for quantified area). n=6-8 each; t-test; ns, not significant. (O) Chromogenic immunostainings on coronal brain sections (bregma -1.3) for NeuN$^+$ neurons (age 16 weeks). Scale bar, 100 µm. (P) Unaltered number of NeuN$^+$ neocortical neurons in *NC-Nrg1* mice compared to controls (*Stop-Nrg1*, *NEX-Cre).* Semi-automated quantification of somatosensory cortex columns (width 600 µm) using watershed thresholded images. n=4-7 each; t-test; ns, not significant. (Q, R) as in (O, P); age 52 weeks.
