# Supplementary material for: Extra-Synaptic Functions of NRG1 Type I Signaling in Hippocampal γ-Oscillations
Source: Schizophr Bull. 2026 Oct 30;52(6):sbag168. doi: 10.1093/schbul/sbag168 (PMC13720722; doi:10.1093/schbul/sbag168)
Supplement: Supplementary file 1 [file supplementary_files_sbag168.zip › Supplementary_Figure_5_sbag168.pdf]

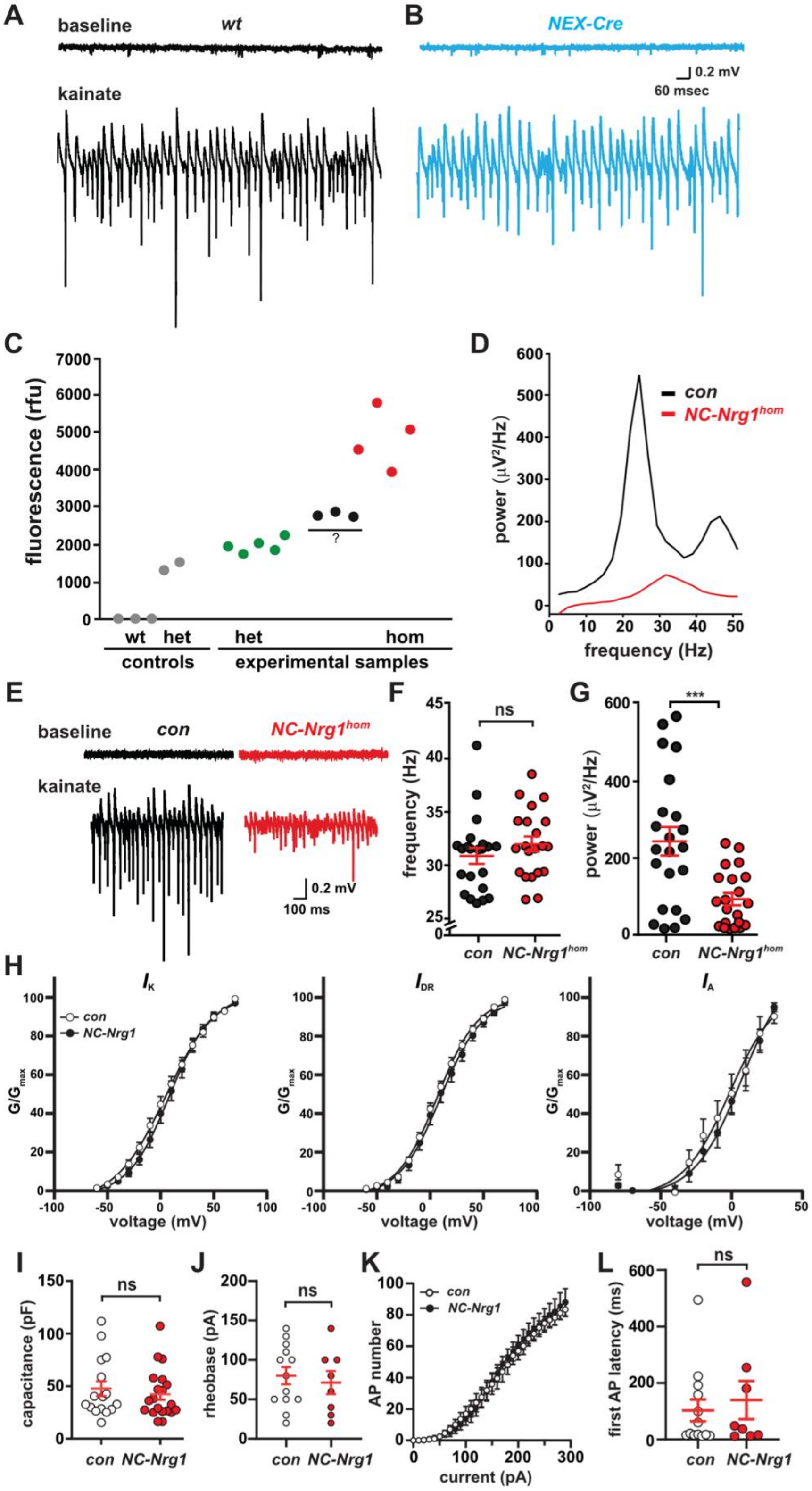

Suppl. Fig. 5: NC-Nrg1 mice exhibit reduced γ-oscillation power but preserved CA3 PV$^+$ interneuron excitability.

(A-B) Representative recordings from the hippocampal CA3 region of wildtype (*wt*) and *NEX-Cre* driver mice at P30 before (baseline) and during kainate-induction of γ-oscillations. (C) qPCR to identify

homozygous *STOP-Nrg1*NEX-Cre* mice. Only mice with the highest fluorescence values (rfu; red circles) were considered homozygous and used for analysis. wt and heterozygous nice genotyped by standard PCR, were used as controls. (D) Representative power spectra of γ-oscillations in controls and transgenic mice homozygous for the *Stop-Nrg1* cassette (*NC-Nrg1^{hom}*). (E) Representative traces of γ-oscillations recorded in the CA3 pyramidal layer in controls and *NC-Nrg1^{hom}* mice. (F) The γ-oscillation frequency was not changed, but (G) averaged power of γ-oscillations was reduced in *NC-Nrg1^{hom}* mice compared to controls. See supplementary table 1 for statistics. (H) Normalized conductance–voltage (G/G_{max}) relationships for I_K (left), I_{DR} (middle), and I_A (right) as a function of voltage (mV) in CA3 PV^+ interneurons from control (open symbols) and *NC-Nrg1* mice (closed symbols). Data were fitted with a Boltzmann function (n= 16-20, 5 mice each). (I) Scatter plot of cell capacitance (pF) in CA3 PV^+ interneurons (n=16-20). (J) Scatter plot of rheobase current (pA) in CA3 PV^+ interneurons (n=8-13). (K) AP number as a function of injected current (pA) in CA3 PV^+ interneurons (n=8-13). (L) Scatter plot of the latency to first AP (ms) in response to rheobase current injection in CA3 PV^+ interneurons (n=8-13). All plots are presented as mean ± SEM. Statistical comparisons were performed using Student’s unpaired *t*-test (ns, not significant).
