# Supplementary material for: Extra-Synaptic Functions of NRG1 Type I Signaling in Hippocampal γ-Oscillations
Source: Schizophr Bull. 2026 Oct 30;52(6):sbag168. doi: 10.1093/schbul/sbag168 (PMC13720722; doi:10.1093/schbul/sbag168)
Supplement: Supplementary file 1 [file supplementary_files_sbag168.zip › Supplementary_Figure_6_sbag168.pdf]

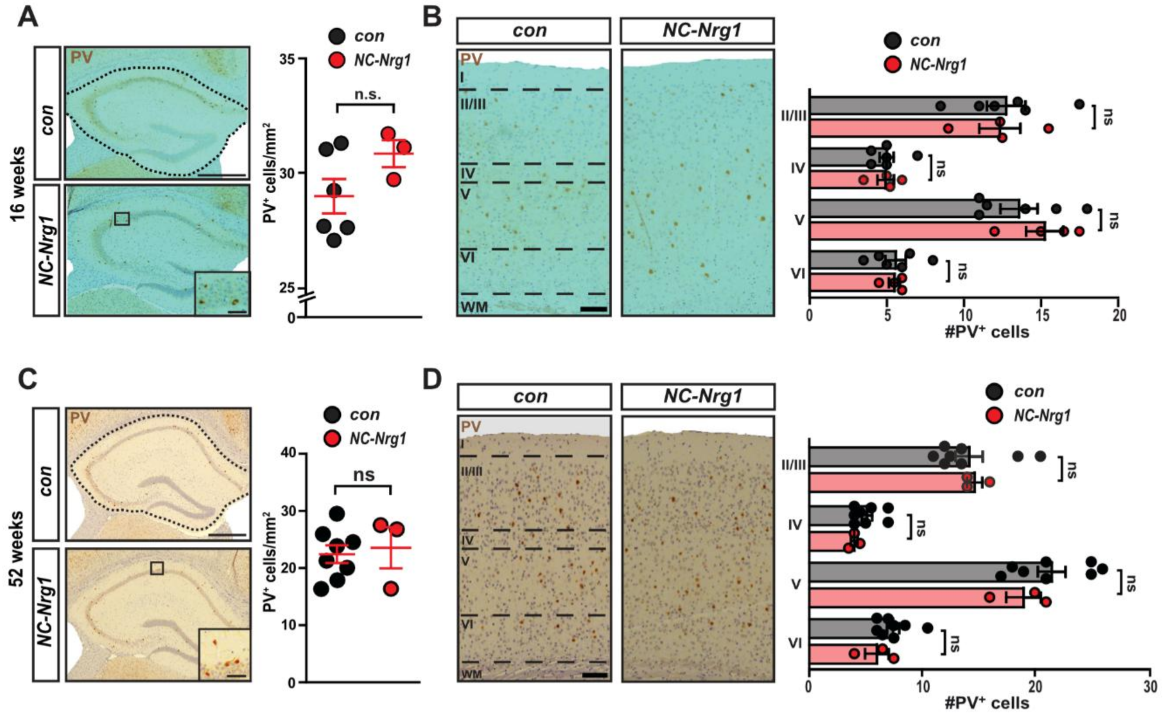

Suppl. Fig. 6: Numbers and cortical positions of PV$^+$ interneurons are unaltered in hippocampus and neocortex of adult and aged *NC-Nrg1* mice.

(A, B) Chromogenic immunostainings for PV on coronal brain sections (age 16 weeks; bregma -1.3). (A, left) Dashed line indicates quantified area in hippocampus. Scale bars, 500 µm, 50 µm (inset). (right) Quantification of PV$^+$ interneurons in *NC-Nrg1* mice and controls (*Stop-Nrg1*, *NEX-Cre*). n=3-6 each; t-test, ns, not significant. (B, left) Neocortical layers are marked by dashed lines. Scale bar, 100 µm. I–VI, cortical layers; WM, white matter. (right) Quantification of PV$^+$ interneurons in somatosensory cortex (column width 600 µm) of *NC-Nrg1 mice* and controls (*Stop-Nrg1, NEX-Cre*). n=3-6 each; ns, p>0.05; t-test. (C, D) Chromogenic immunostainings for PV on coronal brain sections (age 52 weeks; bregma 1.7). (C, left) Dashed line marks quantified area in hippocampus. Scale bars, 500 µm, 50 µm (inset). (right) Quantification of PV$^+$ interneurons in *NC-Nrg1* mice and controls (*Stop-Nrg1*, *NEX-Cre*). n=3-8 each; t-test, ns, not significant. (D, left) Cortical layers are marked by dashed lines. Scale bar, 100 µm. I–VI, cortical layers; WM, white matter. (right) Quantification of PV$^+$ interneurons in somatosensory cortex (column width 600 µm) of *NC-Nrg1 mice* and controls (*Stop-Nrg1, NEX-Cre*). n=3-8 each; t-test; ns, not significant.
