# Supplementary material for: Extra-Synaptic Functions of NRG1 Type I Signaling in Hippocampal γ-Oscillations
Source: Schizophr Bull. 2026 Oct 30;52(6):sbag168. doi: 10.1093/schbul/sbag168 (PMC13720722; doi:10.1093/schbul/sbag168)
Supplement: Supplementary file 1 [file supplementary_files_sbag168.zip › Supplementary_Figure_7_sbag168.pdf]

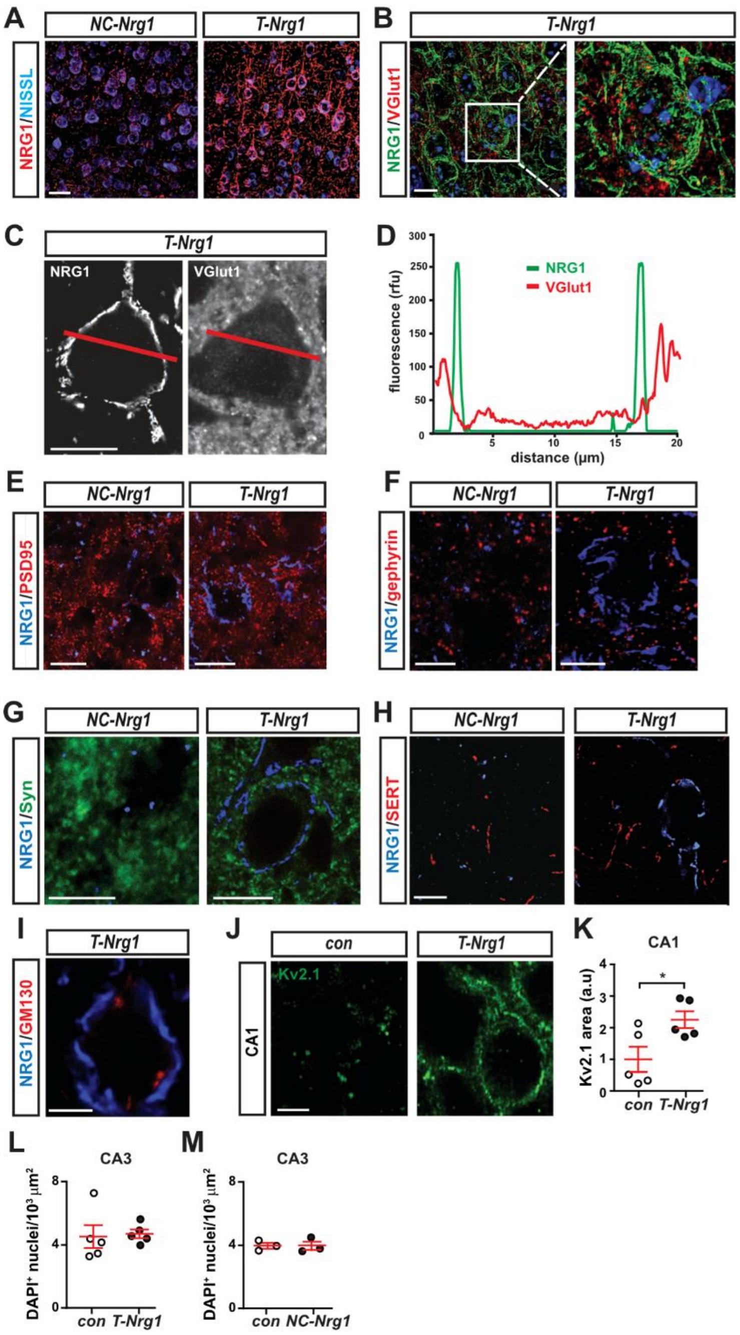

Suppl. Fig. 7: Extra-synaptic NRG1 type I expression in glutamatergic neurons of *T-Nrg1* and *NC-Nrg1* mice.

(A) Moderate NRG1 type I overexpression in the neocortex of *NC-Nrg1* mice compared to massive overexpression in *T-Nrg1* mice. Fluorescent immunostainings of coronal brain sections (age 8-9 weeks) with an antibody directed against the C-terminal domain of NRG1. Scale bar, 25 µm. (B) Fluorescent immunostaining on coronal brain section from *T-Nrg1* mouse (age 8-9 weeks) revealed no colocalization of NRG1 type I (C-terminal domain) with presynaptic marker VGlut1. Scale bar, 10 µm. (C) Absence of colocalization of NRG1 type I with VGlut1 in the somatosensory cortex of *T-Nrg1* mice (age 6 weeks). Fluorescent co-immunostaining (shown are individual channels for the C-terminal domain of NRG1 and VGlut1) of a layer V projection neuron with massive NRG1 type I overexpression. Scale bar, 10 µm. (D) Line scan (indicated by red line in C) was performed using the plot profile function of ImageJ. (E, F) No overlap of NRG1 type I puncta with postsynaptic markers for glutamatergic (PSD95) and GABAergic

synapses (Gephyrin) in somatosensory cortex of *NC-Nrg1* and *T-Nrg1* mice. Scale bars, 10 µm (E), 5 µm (F). (G, H) Fluorescent immunostainings on coronal brain sections revealed absence of colocalization of NRG1 type I (C-terminal domain) with presynaptic marker synaptophysin (G) and serotonergic marker SERT (H) in the cortex of *NC-Nrg1* and *T-Nrg1* mice. Scale bars, 10 µm. (I) Absence of NRG1 type I expression from Golgi complex in *T-Nrg1* mice revealed by co-immunostaining for Golgi marker GM130 on coronal brain sections (age 6 weeks). Scale bar, 5 µm. (J) Fluorescent immunostainings for Kv2.1 on coronal brain sections from control and *T-Nrg1* mice (age 10 weeks). Scalebar, 5 µm. (K) Quantification of Kv2.1$^+$ area fraction in CA1 pyramidal cell layer. (L, M) Number of DAPI$^+$ nuclei in hippocampal CA3 region of *T-Nrg1* (L) and *NC-Nrg1* (M) mice.
