# Supplementary material for: Extra-Synaptic Functions of NRG1 Type I Signaling in Hippocampal γ-Oscillations
Source: Schizophr Bull. 2026 Oct 30;52(6):sbag168. doi: 10.1093/schbul/sbag168 (PMC13720722; doi:10.1093/schbul/sbag168)
Supplement: Supplementary file 1 [file supplementary_files_sbag168.zip › Supplementary_Figure_8_sbag168.pdf]

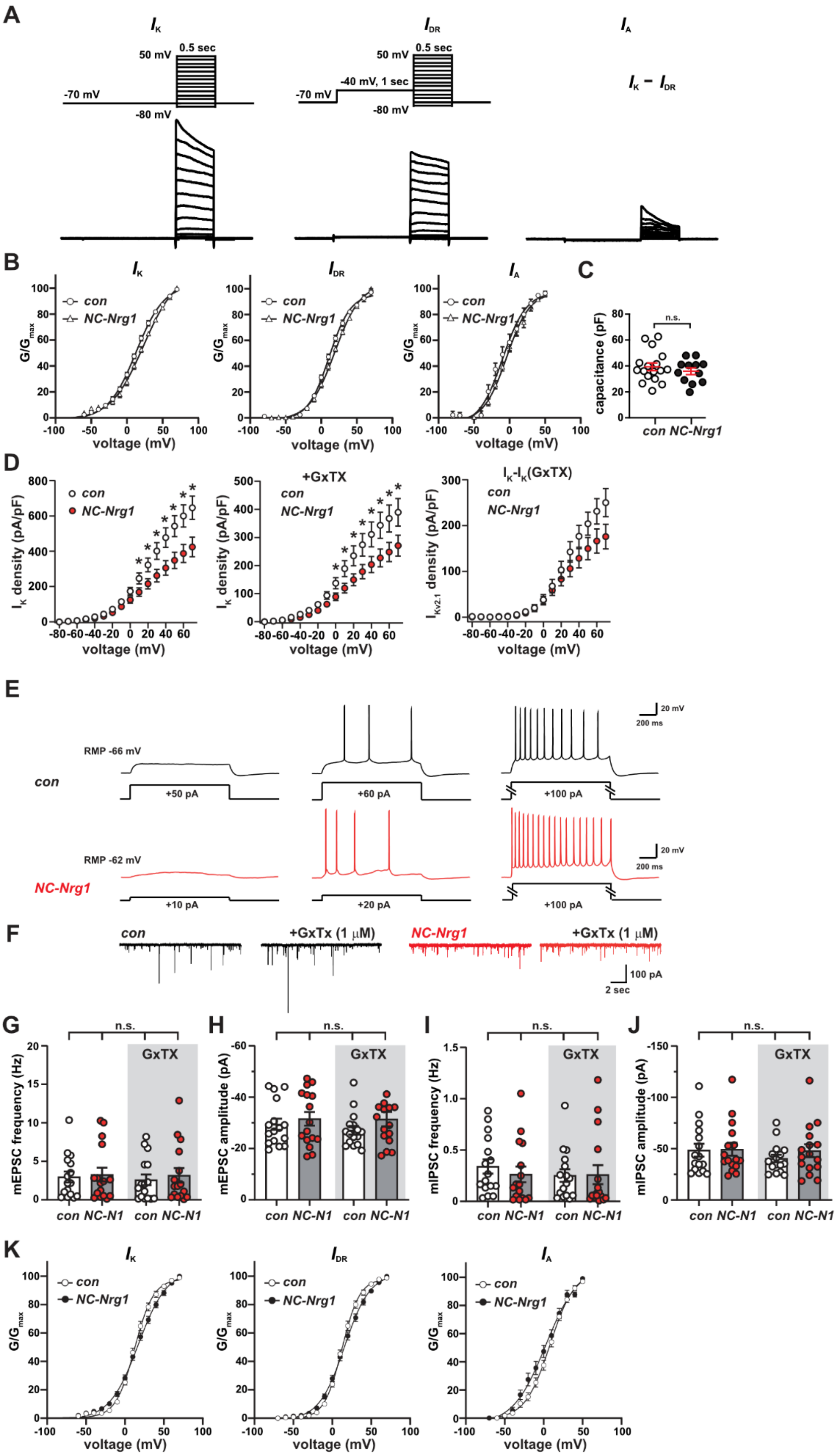
A
I_K
I_DR
I_A
50 mV
0.5 sec
-70 mV
-80 mV
-40 mV, 1 sec
I_K − I_DR
B
C
con
NC-Nrg1
G/G_max
voltage (mV)
capacitance (pF)
n.s.
D
+GxTX
I_K-I_K(GxTX)
I_K density (pA/pF)
I_Kv2.1 density (pA/pF)
E
RMP -66 mV
RMP -62 mV
+50 pA
+60 pA
+100 pA
+10 pA
+20 pA
20 mV
200 ms
F
+GxTx (1 μM)
100 pA
2 sec
G
H
I
J
GxTX
mEPSC frequency (Hz)
mEPSC amplitude (pA)
mIPSC frequency (Hz)
mIPSC amplitude (pA)
con NC-N1 con NC-N1
K

Suppl. Fig. 8: Potassium current properties and synaptic activity

(A) Voltage-clamp protocols and representative current traces for isolating K^+ current components, I_K, I_{DR} and I_A, in CA3 pyramidal neurons. (B) Normalized conductance–voltage (G/G_{max}) relationships for I_K (left), I_{DR} (middle), and I_A (right) as a function of voltage (mV) in CA3 pyramidal neurons from con (circle) and *NC-Nrg1* mice (triangle) (n=12-17; 4 mice each). Data were fitted with a Boltzmann equation. (C) Scatter plot of cell capacitance (pF) in CA3 pyramidal neurons (n=12-17; 4 mice each). (D) Current-voltage relationship of K^+ currents in CA3 pyramidal neurons before (I_K; left panel) and during (+GxTX; middle panel) GxTX treatment, and isolated GxTX sensitive K^+ current (I_K - I_{K+GxTX}; right panel). GxTX sensitive K^+ currents were isolated by subtracting total I_K currents with I_{K+GxTX} (n=21-27; 6 mice each). Current density (pA/pF) values of I_K and I_{K+GxTX} were significantly reduced in CA3 pyramidal neurons from *NC-Nrg1* mice compared to control (*, $P < 0.05$; Student's unpaired t-test; n=21 for control and n=27 for *NC-Nrg1*). (E) Representative membrane potential recordings from CA3 pyramidal neurons of con (RMP = −63 mV) and *NC-Nrg1* mice (RMP = −62 mV) in response to sub-threshold (−50 pA), rheobase (+60 pA/+100 pA), and 2× rheobase (+120 pA/+200 pA) current injections. Scale bars: 20 mV, 200 ms. (F) Representative mEPSC and mIPSC traces of control (black line) and NC-Nrg1 (red line) recorded in hippocampal slice CA3 pyramidal neuron (left) in presence or absence with 1 µM GxTX (right). Scale bars; 2 sec and **100 pA**. (G) mEPSC frequency of control (n=16; 4 mice) and NC-Nrg1 mice (n=16; 4 mice) before and during GxTX treatment. (H) mEPSC amplitude of control and NC-Nrg1 mice (n=16) with or without GxTX treatment. (I) mIPSC frequency of control and NC-Nrg1 mice (n=16) before and during GxTX treatment. (J) mIPSC amplitude of control and NC-Nrg1 mice with or without GxTX treatment (n=16). Data are presented as mean ± SEM. Statistical comparisons were performed using Student's *t*-test and one-way ANOVA comparisons test (ns, not significant). (K) Normalized conductance–voltage (G/G_{max}) relationships for I_K, I_{DR}, and I_A as a function of voltage (mV) in CA1 pyramidal neurons from con (open circle) and *NC-Nrg1* mice (closed circle). Data were fitted with a Boltzmann function (n= 21-24; 6 mice each).
