# Supplementary material for: Extra-Synaptic Functions of NRG1 Type I Signaling in Hippocampal γ-Oscillations
Source: Schizophr Bull. 2026 Oct 30;52(6):sbag168. doi: 10.1093/schbul/sbag168 (PMC13720722; doi:10.1093/schbul/sbag168)
Supplement: Supplementary file 1 [file supplementary_files_sbag168.zip › Supplementary_Methods_sbag168.docx]

**Supplementary Methods**

**Fluorescence in situ hybridization (FISH)**

The RNAscope Multiplex Fluorescent v2 Assay (Advanced Cell Diagnostics) was performed on mouse hippocampal primary neurons cultured on poly-L-lysine (PLL)-coated coverslips, including untreated controls and KCl-treated cultures. Neurons were fixed in 4% PFA for 30 min at RT, followed by dehydration in a graded ethanol series (50%, 70%, 100%). Coverslips were rehydrated through decreasing ethanol concentrations (100%, 70%, 50%) and washed in 1x PBS. Cells were permeabilized using RNAscope Protease III for 10 min at RT.

An RNAscope probe targeting *Nrg1* mRNA (Cat No. 418181-C3) was hybridized according to the manufacturer’s instructions at 40°C, followed by sequential signal amplification and fluorescent labeling. Nuclei were counterstained with DAPI. Coverslips were mounted using Aqua-Poly/Mount (Polysciences, Inc.) and stored at 4°C until imaging.

Coronal brain sections were processed according to the manufacturer’s instructions for fresh-frozen tissue using RNAscope™ Multiplex Fluorescent Reagent Kit v2. Sections were removed from -80°C, kept for 30 min at 4°C, and baked at 60°C. Sections were post-fixed with 4% PFA and dehydrated in a graded ethanol series (50%, 70%, 100%). After a short incubation in H_2_O_2_, and antigen retrieval according to the manufacturer’s instructions, sections were permeabilized using RNAscope Protease III for 30 min at 40°C. Subsequently, a 2h incubation with an RNAscope probe mix targeting Nrg1 type I (RNAscope™ Probe - Mm-Nrg1-O8, Catalog No.: 854561-C2, targeting 11-887 of NM_001364421.2; probe binding region includes 5' UTR) and Nrg1 type III (RNAscope™ Probe - Mm-Nrg1-O5. Catalog No.: 870451-C3, targeting 34 - 1171 of NM_001364421.2; probe binding region includes 5' UTR) mRNA was performed. After sequential signal amplification and fluorescent labeling, nuclei were counterstained with Hoechst, coverslips were mounted using Aqua-Poly/Mount (Polysciences, Inc.) and stored at 4°C until imaging. Samples were imaged using confocal microscopy (Zeiss LSM 880 Airyscan confocal microscope).
