# Supplementary material for: Extra-Synaptic Functions of NRG1 Type I Signaling in Hippocampal γ-Oscillations
Source: Schizophr Bull. 2026 Oct 30;52(6):sbag168. doi: 10.1093/schbul/sbag168 (PMC13720722; doi:10.1093/schbul/sbag168)
Supplement: Supplementary file 1 [file supplementary_files_sbag168.zip › Supplementary_Table_1_sbag168.docx]

**Supplementary Table 1. Statistics γ-oscillations**

|  |  | **Experimental Group** | **Average ± SEM** | **P Value** | **Statistical Test** | **N** |
| --- | --- | --- | --- | --- | --- | --- |
| Fig. 3D, E | CTL vs Nrg1 Heterozygous OE Frequency (Hz) | con | 31.42 ± 0.6473, n=18 | 0.1624  ns | Student’s t-test | 3 |
|  |  | NC-Nrg1 | 30.13 ± 0.628, n=18 |  | Student’s t-test | 3 |
|  | CTL vs Nrg1 Heterozygous OE Average Power (μV^2^/Hz) | con | 951.2 ± 176, n=18 | 0.0037  ** | Student’s t-test | 3 |
|  |  | NC-Nrg1 | 383 ± 47.44, n=18 |  | Student’s t-test | 3 |
| Fig. 3G, H | CTL vs NEX_Cre  Frequency (Hz) | WT | 34.03 ± 0.9699, n=11 | 0.2119  ns | Student’s t-test | 2 |
|  |  | NEX-Cre | 32.44 ± 0.7648, n=12 |  | Student’s t-test | 2 |
|  | CTL vs NEX_Cre  Average Power (μV^2^/Hz) | WT | 626.5 ± 134.9, n=11 | 0.4365  ns | Student’s t-test | 2 |
|  |  | NEX-Cre | 781.1 ± 140.8, n=10 |  | Student’s t-test | 2 |
| Fig. 3J, K | CTL vs Nrg1 Heterozygous OE Frequency (Hz) | con | 40.11 ± 1.154, n=19 | 0.1582  ns | Student’s t-test | 4 |
|  |  | NC-Nrg1 | 42.28 ± 0.9983, n=23 |  | Student’s t-test | 4 |
|  |  | con + PD158780 | 39.45 ± 1.038, n=19 | 0.3571  ns | Student’s t-test | 4 |
|  |  |  |  | 0.6799  ns | Student’s t-test |  |
|  |  | NC-Nrg1 + PD158780 | 40.02 ± 0.9014, n=23 |  |  | 4 |
|  |  |  |  | 0.0973  ns | Student’s t-test |  |
|  | CTL vs Nrg1 Heterozygous OE Average Power (μV^2^/Hz) | con | 386.8 ± 58.97, n=19 | 0.0166  * | Student’s t-test | 4 |
|  |  | NC-Nrg1 | 210.5 ± 41.8,1 n=23 |  | Student’s t-test | 4 |
|  |  | con + PD158780 | 579.1 ± 87.23, n=19 | 0.0030  ** | Student’s t-test | 4 |
|  |  |  |  | 0.6067  ns | Student’s t-test |  |
|  |  | NC-Nrg1 + PD158780 | 508.5 ± 100.5, n=23 |  |  | 4 |
|  |  |  |  | 0.0004  *** | Student’s t-test |  |
| Suppl. Fig. 5F, G | CTL vs Nrg1 Homozygous OE Frequency (Hz) | con | 30.88 ± 0.7519, n=22 | 0.2857  ns | Student’s t-test | 4 |
|  |  | NC-Nrg1hom | 31.99 ± 0.6982, n=21 |  | Student’s t-test | 4 |
|  | CTL vs Nrg1 Homozygous OE Average Power (μV^2^/Hz) | con | 243.9 ± 37.11, n=22 | 0.0007  *** | Student’s t-test | 4 |
|  |  | NC-Nrg1hom | 93.12 ± 16.07, n=21 |  | Student’s t-test | 4 |
